# Molecular Insights into the Breast and Prostate Cancer Cells in Response to the Change of Extracellular Zinc

**DOI:** 10.1155/2024/9925970

**Published:** 2024-01-12

**Authors:** Shital K. Barman, Monokesh K. Sen, David A. Mahns, Ming J. Wu, Chandra S. Malladi

**Affiliations:** ^1^School of Science, Western Sydney University, Locked Bag 1797, Penrith, NSW 2751, Australia; ^2^Charles Perkins Centre, School of Medical Sciences, Faculty of Medicine and Health, The University of Sydney, Camperdown 2006, NSW, Australia; ^3^School of Medicine, Western Sydney University, Locked Bag 1797, Penrith, NSW 2751, Australia; ^4^Proteomics and Lipidomics Lab, School of Medicine, Western Sydney University, Locked Bag 1797, Penrith, NSW 2751, Australia

## Abstract

Zinc dyshomeostasis is manifested in breast and prostate cancer cells. This study attempted to uncover the molecular details prodded by the change of extracellular zinc by employing a panel of normal and cancerous breast and prostate cell lines coupled with the top-down proteomics with two-dimensional gel electrophoresis followed by liquid chromatography-tandem mass spectrometry. The protein samples were generated from MCF-7 breast cancer cells, MCF10A normal breast cells, PC3 prostate cancer cells, and RWPE-1 normal prostate cells with or without exogenous zinc exposure in a time course (*T*_0_ and *T*_120_). By comparing the cancer cells vs respective normal epithelial cells without zinc treatment (*T*_0_), differentially expressed proteins (23 upregulated and 18 downregulated in MCF-7 cells; 14 upregulated and 30 downregulated in PC3 cells) were identified, which provides insights into the intrinsic differences of breast and prostate cancer cells. The dynamic protein landscapes in the cancer cells prodded by the extracellular zinc treatment reveal the potential roles of the identified zinc-responsive proteins (e.g., triosephosphate isomerase, S100A13, tumour proteins hD53 and hD54, and tumour suppressor prohibitin) in breast and prostate cancers. This study, for the first time, simultaneously investigated the two kinds of cancer cells related to zinc dyshomeostasis, and the findings shed light on the molecular understanding of the breast and prostate cancer cells in response to extracellular zinc variation.

## 1. Introduction

Zinc (Zn^2+^) is essential to life. It functions in the cell as a cofactor for well over 300 enzymes and as a structural component for approximately 10% of the human proteome (∼3000 proteins) [1]. Consequently, the cell has developed an elaborate molecular network over the extensive evolutionary timeline to maintain zinc homeostasis. Any disruption of such a network may lead to zinc dyshomeostasis, resulting in health problems such as cancers. Breast cancer is the most common malignancy in females worldwide [2, 3], and prostate cancer in males is the second and fifth highest in incidence and mortality, respectively [2]. Both breast and prostate cancers are associated with intracellular zinc dysregulation. Breast cancer cells exhibit elevated intracellular zinc levels compared to their normal epithelial cells [4], while prostate cancer cells show decreased intracellular zinc levels compared to their normal counterparts [5]. Such diametrically opposite zinc profiles of breast and prostate cancer cells provide an avenue for understanding the role of zinc in these two types of cancer cells.

It is well documented that cellular zinc homeostasis is maintained by Zrt/Irt-like protein (ZIP), Zn^2+^ transporter (ZnT), and metallothionein (MT) [6–8]. ZIP family contains 14 members, ZIP1-14 encoded by *SLC39A1-14*. They increase the cytoplasmic zinc level by importing zinc from the extracellular space or the intracellular organelles/vesicles into the cytoplasm. In contrast, ZnT family, which has 10 members as ZnT1-10 encoded by *SLC30A1-10*, reduces cytoplasmic zinc by exporting cytoplasmic zinc out of the cell or into the lumens of intracellular organelles. MT family buffers cytoplasmic zinc to maintain zinc homeostasis [9]. The elevated accumulation of intracellular zinc in breast cancer cells or the reduced intracellular zinc in prostate cancer cells is associated with the dynamic expression of ZIP, ZnT, and MT [10, 11]. Previous studies demonstrated that the extracellular zinc exposure resulted in the elevation of intracellular zinc [12–15]. Therefore, this study attempts to prod the molecular machinery for zinc homeostasis into action by applying the extracellular zinc exposure and then uncover the dynamic changes by the proteomic approach. As intracellular zinc levels are fluctuating in the cells of living human beings, the dynamic changes in the proteomes of breast and prostate cancer cells are indeed relevant to our understanding of the zinc homeostasis in cancer cells.

Proteomics, complementary to genomics, is an established and essential platform for cancer research [16]. Proteomic analysis on breast and prostate cancer tissues or cell lines or biological fluids from the cancerous individuals was employed in previous studies for the discovery and validation of the predictive, diagnostic, and prognostic markers [17–26]. Differential protein profiles have been generated by the proteomics approach employing normal tissues and malignant tissues of low- or high-grade cancers [23]. Comparative proteome analysis reveals changes in the proteins associated with metabolism [20, 27], drug resistance, and metastasis of breast and prostate cancer cells [28, 29]. However, the proteomic profiling has not been simultaneously carried out thus far in normal and cancerous cells of breast and prostate with or without extracellular zinc manipulation. Proteomic insights might be gained by investigating these two types of cancer cells with extracellular zinc manipulation which could prod the cells to action in response to the change of extracellular zinc.

In this study, the top-down proteomic analysis, by two-dimensional gel electrophoresis (2-DE) coupled with liquid chromatography-tandem mass spectrometry (LC-MS/MS), was systematically carried out on MCF-7 breast cancer cells and MCF10A normal breast epithelial cells, PC3 prostate cancer cells, and RWPE-1 normal prostatic epithelial cells, with and without exogenous zinc exposure. The following comparisons were performed in the data analysis: (1) the cancer cells vs the corresponding normal cells without zinc treatment (*T*_0_) including MCF-7 cells vs MCF10A cells and PC3 cells vs RWPE-1 cells, (2) the cancer cells vs the respective normal cells with exogenous zinc treatment for 120 min (*T*_120_) including MCF-7 cells *T*_120_ vs MCF10A cells *T*_120_ and PC3 cells *T*_120_ vs RWPE-1 cells *T*_120_, (3) comparison of cancer cells between *T*_120_ and *T*_0_ including MCF-7 cells *T*_120_ vs MCF-7 cells *T*_0_ and PC3 cells *T*_120_ vs PC3 cells *T*_0_, and (4) comparison of the normal cells between *T*_120_ and *T*_0_ including MCF10A cells *T*_120_ vs MCF10A cells *T*_0_ and RWPE-1 cells *T*_120_ vs RWPE-1 cells *T*_0_. Such detailed comparative analyses revealed differential protein expression profiles of breast and prostate cells in the context of with or without extracellular zinc treatment, which provides significant insights and enhances our understanding of the breast and prostate cancer cells in response to extracellular zinc variation.

## 2. Materials and Methods

All the chemicals and reagents were of the highest purity grade from commercial providers as indicated in the methods. All the cell lines were purchased from American Type Culture Collection (ATCC, USA).

### 2.1. Cell Culture and Protein Extraction from ZnSO_4_-Treated and Untreated Cells

Breast cells (MCF10A, MCF-7) and prostate cells (RWPE-1, PC3) were cultured in their standard growth media and condition described previously [10]. According to the formulations of the media used here and the available data for the zinc contents in foetal bovine serum [30] and horse serum [31, 32], the base level of zinc for the complete DMEM and RPMI 1640 media is approximately 5 *μ*M, that for the complete DMEM/F12 is approximately 2 *μ*M, and that for the complete keratinocyte serum free medium is 0.5 *μ*M. The mild cytotoxic dosage of ZnSO_4_ for each cell line was determined by treating the cells with the individual dosages of ZnSO_4_ including 0, 20, 50, 100, 150, 200, 250, 300, 350, 400, and 500 *μ*M as described in the previous studies [10, 14]. Each dosage was the final concentration of ZnSO_4_, which was carried out by adding 10 *μ*L of the 20X ZnSO_4_ stock to the culture well containing 7000 cells in 190 *μ*L medium (the final volume per well was 200 *μ*L). The mild cytotoxic dosage for ZnSO_4_ was defined as the dosage which resulted in above 70%–85% cell viability at the end of 2 h zinc sulfate treatment. In this study, we used mild cytotoxic ZnSO_4_ dosages of MCF-7 (320 *μ*M), MCF10A (195.5 *μ*M), PC3 (110 *μ*M), and RWPE-1 (186.88 *μ*M) cells for zinc treatment in proteomic analysis. The rationale for selecting the mild cytotoxic dosages of ZnSO_4_ is to obtain the datasets on differentially expressed proteins prodded by the dosages without severely compromising the overall health of the cells in the culturing flasks of this study. The viability of cells between 70% and 85% is ideal here, which allows the findings to be relevant to the physiological state of the cells and provides maximum data possible. MCF-7, MCF10A, PC3, and RWPE-1 cells were grown in 75 cm^2^ flasks until achieving ∼80% confluency and then the spent medium was aspirated and replaced with 11.9 mL of complete medium. ZnSO_4_ at 120x stock concentration of each dosage for each cell line was prepared in sterile Milli-Q H_2_O (Milli-Q® Advantage A10 Water Purification System, Merck, Australia). The cells were treated with 100 *μ*L of their respective ZnSO_4_ stocks. The control cells were treated with 100 *μ*L of sterile Milli-Q water. The cells were incubated for 120 min (*T*_120_) and then the protein extraction was performed. Each treatment or control has three biological replicates, which means three protein samples for each time point of a given treatment or control. Each protein sample was prepared with three 75 cm^2^ flasks of ∼80% confluence.

Following the completion of incubation period, the medium was discarded, and the cells were washed and collected in 1x phosphate-buffered saline (PBS). The cell pellets were obtained by centrifugation at 350 *g* for 3 min at 4°C and washed with ice cold 1x PBS twice. Finally, the cell pellet was resuspended into 1 mL of ice cold 1x PBS and transferred into sterile 1.5 mL microfuge tubes. The cells were centrifuged at 6000 rpm at 4°C for 5 min and the supernatant was discarded. The cells were snap frozen in liquid nitrogen and stored at −80°C for protein extraction.

150–200 *μ*L of total protein extraction buffer containing 8 M urea (Amresco, Solon, OH, USA), 2 M thiourea (Amresco, Solon, OH, USA), 4% CHAPS (Amresco, Solon, OH, USA), and 1x protease inhibitors (Sigma-Aldrich) was added to each cell pellet in a microfuge tube kept on ice. The cells were then homogenised by using a probe sonicator (Across International, Australia) and centrifuged at 124436 *g* (SW 55 Ti rotor, Beckman Coulter, Indianapolis, IN, USA) at 4°C for 1 h. The supernatants were collected into individual tubes for either immediate analysis or storage at −80°C.

### 2.2. Protein Quantification, Reduction, and Alkylation for 2D Gel Electrophoresis

The protein concentration for each sample was estimated using the EZQ™ protein quantitation kit (Life Technologies, Eugene, OR, USA) according to the manufacturer's instructions. 100 *μ*g of each protein sample was taken in a sterile 0.65 mL microfuge tube. An equal volume of rehydration buffer (containing a mixer of carrier ampholytes (Bio-Lyte, Bio-Rad, Australia) at a final concentration 2% (v/v)) was added to each tube. The sample was then mixed with 2.42 *μ*L of reduction buffer (2 M DTT in 0.2 M TBP) and incubated at 25°C for 1 h on a heating block (Dry Block Heater, Thermoline Scientific, Australia). Following the incubation, 5.1 *μ*L acrylamide (5.6 M) was added to each protein sample for alkylation, vortexed, and incubated at 25°C for further 1 h. The protein samples were then ready for 2D separation.

### 2.3. First Dimension-Isoelectric Focusing (IEF)

The nonlinear 7 cm long immobilised pH gradient (IPG) strips with pH 3–10 gradients were hydrated with 125 *μ*L of the above-treated protein sample (100 *μ*g). The IEF was then carried out by using Protean IEF apparatus (Bio-Rad, USA) with the following program: desalting at 250 V for 15 min, ramping up the voltage to 4000 V by a linear gradient for 2 h, keeping 4000 V constant for a total of 37500 Vh and then terminating the isoelectric focusing or holding at 500 V until the termination. The temperature of the IEF apparatus during isoelectric focusing was 17°C. Upon completion of the isoelectric focusing, the IPG strips were immediately subjected to the second dimension.

### 2.4. Second Dimension-Sodium Dodecyl Sulfate Polyacrylamide Gel Electrophoresis (SDS-PAGE)

The IPG strips from the first dimension were incubated in 130 mM DTT in equilibration buffer for 10 min followed by 10 min alkylation with 350 mM acrylamide at room temperature on a gentle shaker. Instantly, the IPG strip was placed in warm agarose layer over the stacking gel (5%) which was above the resolving gel (12.5% (w/v) acrylamide, 1 mm thick 8.4 × 7 cm). Once the agarose layer was solidified, the electrophoresis was carried out in 1x tris-glycine-SDS running buffer at 90 V and 4°C for 3 h until the tracking bromophenol blue dye reached the bottom of the gel. Finally, the gels were taken out of the glass plates, promptly dipped into the fixatives (10% methanol with 7% acetic acid) for gel fixation, and then stained with colloidal Coomassie Brilliant Blue (cCBB) for 20 h, followed by destaining with 0.5 M NaCl thrice (15 min each). The gels were imaged by FUJI LAS-4000 (GE Healthcare, USA).

### 2.5. Protein Spot Detection and Quantitative Analysis

The protein spots were detected and quantitatively analysed in the gel images by Delta2D (version 4.0.8, DECODON Gmbh, Germany) as described previously [33]. The protein spots in the gel images were quantitatively analysed in cancer cells compared to normal cells with or without zinc treatment. Similarly, the protein spots were analysed in each cell line with or without zinc treatment. In each comparison, the gel images were warped and fused to make master gel using “union fusion.” The spots were then transferred to each image in their group to ensure consistent spot matched (100% matching) in all biological replicates (*n* = 3) in each group. The background-subtracted spot volumes were described as grey values, fold changes, *p* values (*t*-test), and relative standard deviation (RSD). Based on *p* value (*p* < 0.05) and ratio of grey value, the candidate spots were considered for further proteolytic digestion and liquid chromatography-tandem mass spectrometry (LC-MS/MS) to identify the proteins.

### 2.6. Peptide Extraction and LC-MS/MS

The selected protein spots were excised manually and digested with trypsin (Promega, USA) for 8 h at 4°C. The digested protein samples were analysed by LC-MS/MS (Mass Spectrometry Facility, Western Sydney University), using Waters nanoAcquity LC-MS/MSnanoACQUITY UPLC on a Xevo QToF mass spectrometer (Waters, USA) as described previously [34, 35]. The protein identification was conducted employing ProteinLynx Global Server (PLGS) program (version 3.0 Waters Corporation, USA) and the UniProt (*Homo sapiens*, human) database with the following settings: (a) the allowed maximum missed cleavages was set to 1, (b) the allowed false discovery rate was set to 4% and the maximum protein size was set to 280 kDa, (c) the peptide modifications were carbamidomethyl C (fixed) and oxidation M (variable), (d) the minimum fragment per peptide was 3, (e) the minimum peptide per protein was 1, and (f) the minimum fragment per protein was 7. Finally, the identified proteins from each spot by LC-MS/MS had to meet the selection thresholds such as PLGS or protein score ≥200, sequence coverage ≥6%, and matched peptides ≥3.

### 2.7. Literature Mining and Bioinformatics

The identified proteins from both breast and prostate cells were searched in PubMed (https://www.ncbi.nlm.nih.gov/pubmed/), UniProt (https://www.uniprot.org) database, and PANTHER database (https://www.pantherdb.org) to know their expression status, cellular localisation, molecular function, and protein classes.

### 2.8. Analysis of Functional Interactions of the Differentially Expressed Proteins

The functional interactions of the differential expressed proteins in breast cells (MCF-7, MCF10A) and prostate cells (PC3, RWPE-1) were analysed using STRING (https://string-db.org/) following the four comparisons, including cancer vs normal cells without zinc exposure (*T*_0_), cancer vs normal cells with zinc exposure for 120 min (*T*_120_), comparison of cancer cells between *T*_120_ and *T*_0_, and comparison of the normal cells between *T*_120_ and *T*_0_.

## 3. Results

### 3.1. Differentially Expressed Proteins in Breast Cancer Cells (MCF-7) without Zinc Treatment

**Figure 1:**
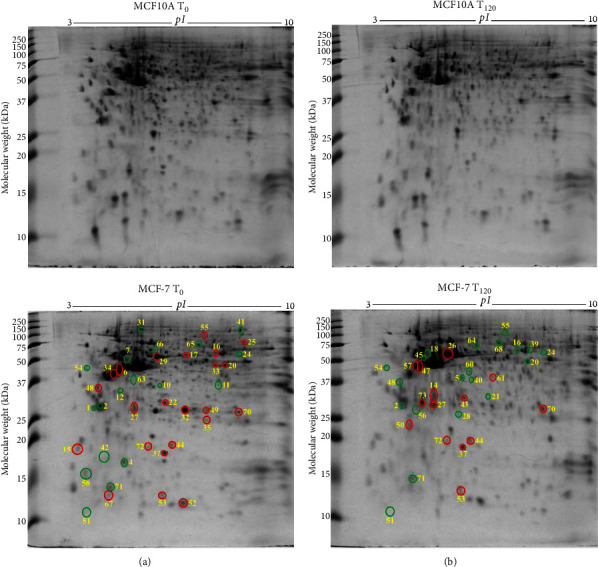
Differentially expressed protein spots in 2-DE gels by comparisons of MCF-7 *T*_0_ vs MCF10A *T*_0_ and MCF-7 *T*_120_ vs MCF10A *T*_120_. (a) Representative 2-DE gel images (in the left panel) of breast normal MCF10A cells (MCF10A *T*_0_) and breast cancer MCF-7 cells (MCF-7 *T*_0_) without zinc exposure (*T*_0_). (b) Representative 2-DE gel images (in the right panel) of breast normal MCF10A cells (MCF10A *T*_120_) and breast cancer MCF-7 cells (MCF-7 *T*_120_) with exogenous zinc exposure for 120 min (*T*_120_). Each protein extract (100 *μ*g) was resolved based on isoelectric point (*pI*) and molecular weight (MW). The differentially expressed protein spots are shown with red circles denoting upregulation and green circles denoting downregulation.

By comparing the protein profiles of MCF-7 breast cancer cells against the normal breast epithelial cells (MCF10A), the differentially expressed proteins in breast cancer cells (MCF-7) were identified. Quantitative analysis of the 2-DE gels by DECODON Delta2D software revealed 23 upregulated (red circles) and 18 downregulated (green circles) protein species in MCF-7 breast cancer cells compared to MCF10A normal breast epithelial cells without exogenous zinc exposure (*T*_0_) (Figure 1(a)). After LC-MS/MS analysis, the identified proteins are listed in Table 1. The proteins such as 14-3-3 protein *σ* (*SFN*), 14-3-3 protein *θ* (*YWHAQ*), and protein S100A2 (*S100A2*) were downregulated in MCF-7 cells, which were shown to have tumour suppression activity by the previous studies [36, 37]. In addition, calcium-binding annexin protein notably annexin A1 (*ANXA1*) is found to be downregulated. The overexpressed proteins which are associated with breast cancer cell progression and invasion include *α*-smooth muscle actin *α*2 (*ACTA2*), cytochrome b5 type B (*CYB5B*), D-3-phosphoglycerate dehydrogenase (*PHGDH*), dihydrolipoamide S-succinyltransferase (*DLST*), elongation factor Tu (*TUFM*), F-actin-capping protein subunit *β* (*CAPZB*), FUBP1 (*FUBP1*), glutathione S-transferase Mu 3 (*GSTM3*), glutathione synthetase (*GSS*), heterogeneous nuclear ribonucleoproteins C1/C2 (*HNRNPC*), high mobility group protein B1 (*HMGB1*), histone H4 (*HIST1H4J*), nucleoside diphosphate kinase (*NME*), proliferating cell nuclear antigen (*PCNA*), peroxiredoxin 6 (*PRDX6*), protein S100A13 (*S100A13*), radixin (*RDX*), triosephosphate isomerase (*TPI1*), and tumour protein D53 (*TPD52L1*).

Based on the molecular functions as per literature survey and UniProt database, those 41 differentially expressed proteins were classified into three prominent groups, including catalytic enzymes (26%), metal ion binding proteins (16%), and molecular chaperones (11%) (Supplementary Figure 1a). PANTHER database-based protein classification agrees with the molecular function-based classification as catalytic enzyme (33%) and calcium binding-protein classes (13%) are the prominent ones (Supplementary Figure 1b). The subcellular localisation classification showed that those proteins are in the cytoplasm (39%), nucleus (22%), and mitochondrion (11%) (Supplementary Figure 1c).

### 3.2. Differentially Expressed Proteins in MCF-7 Breast Cancer Cells Compared to MCF10A Normal Breast Epithelial Cells following Exogenous Zinc Exposure

The extracellular zinc exposure resulted in 20 downregulated (green circle) and 14 upregulated (red circle) protein spots (Figure 1(b)) in MCF-7 breast cancer cells compared to MCF10A normal breast epithelial cells. Tumour suppressor 14-3-3 protein *θ* (*YWHAQ*) and serpin B5 (*SERPINB5*) were downregulated (Table 1). The suppressed proteins, including D-3-phosphoglycerate dehydrogenase (*PHGDH*), elongation factor Tu (*TUFM*), adenylosuccinate lyase (*ADSL*), inosine-5′-monophosphate dehydrogenase (*IMPDH*), L-lactate dehydrogenase B chain (*LDHB*), and perilipin (*PLIN*), are related to catalytic activity (Table 1). The overexpressed proteins, such as cathepsin D (*CTSD*), glutathione S-transferase Mu 3 (*GSTM3*), NADH dehydrogenase (ubiquinone) iron-sulfur protein 3 (*NDUFS3*), actin *γ* (*ACTG2*), protein S100A13 (*S100A13*), 40S ribosomal protein SA (*RPSA*), triosephosphate isomerase (*TPI1*), tumour protein D53 (*TPD52L1*), and tumour protein D54 (*TPD52L2*), are associated with cellular structure, cell proliferation, and metastasis (Table 1). The differentially expressed 34 proteins were classified into 38% catalytic, 14% structural, and 8% signalling proteins based on molecular function according to literature survey and UniProt database (Supplementary Figure 2a). PANTHER database-based classification demonstrated three prominent groups including catalytic enzyme (34%), cytoskeletal proteins (11%), and translational proteins (11%) (Supplementary Figure 2b). The proteins showed their subcellular localisation in the cytoplasm (48%), nucleus (18%), and mitochondrion (12%) (Supplementary Figure 2c).

### 3.3. Differentially Expressed Proteins in MCF-7 Breast Cancer Cells with Exogenous Zinc Exposure Compared to MCF-7 Cells without Zinc Exposure

**Figure 2:**
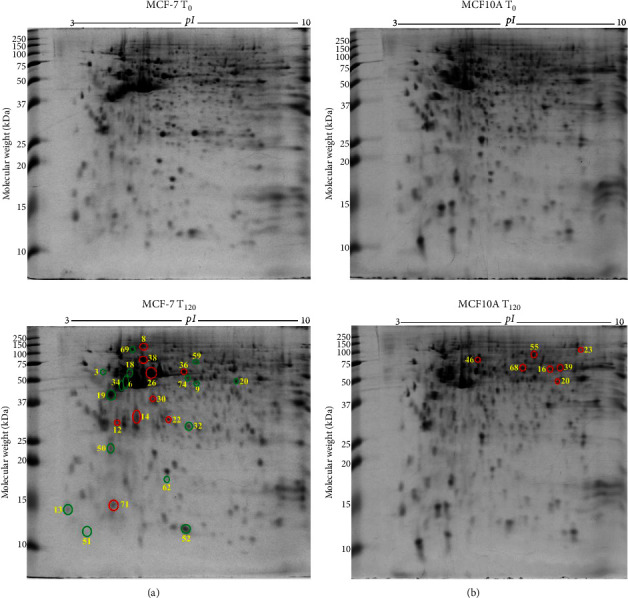
Differentially expressed protein spots in 2-DE gels by comparisons of MCF-7 *T*_120_ vs MCF-7 *T*_0_ and MCF10A *T*_120_ vs MCF10A *T*_0_. (a) Representative 2-DE gel images (in the left panel) of breast cancer MCF-7 cells without zinc (MCF-7 *T*_0_) and with exogenous zinc exposure for 120 min (MCF-7 *T*_120_). (b) Representative 2-DE gel images (in the right panel) of breast normal MCF10A cells without zinc (MCF10A *T*_0_) and with exogenous zinc exposure for 120 min (MCF10A *T*_120_). Each protein extract (100 *μ*g) extract was resolved based on isoelectric point (*pI*) and molecular weight (MW). The differentially expressed protein spots are shown with red circles denoting upregulation and green circles denoting downregulation.

MCF-7 breast cancer cells demonstrated 16 downregulated (green circle) and 9 upregulated (red circle) protein spots (Figure 2(a)) following exogenous zinc exposure at *T*_120_ compared to MCF-7 cells at *T*_0_. The downregulated proteins, including *α*-smooth muscle actin 2 (*ACTA2*), adenosylhomocysteinase (*AHCY*), calmodulin 1 (*CALM1*), heterogeneous nuclear ribonucleoproteins C1/C2 (*HNRNPC*), stathmin (*STMN1*), cytochrome c oxidase subunit 6B1 (*COX6B1*), and vesicle amine transport protein 1 (*VAT1*), are related to cancer cell proliferation and migration (Table 1). Tumour suppressor protein S100A2 (*S100A2*) is downregulated under zinc exposure. The overexpressed proteins, including actinin *α*1 isoform (*ACTN1*), annexin A5 (*ANXA5*), cathepsin D (*CTSD*), F-actin-capping protein subunit *β* (*CAPZB*), inorganic pyrophosphatase (*PPA1*), and tubulin *α*1c chain (*TUBA1C*), are related to cellular structure, growth, or cancer cell invasion (Table 1). Stress protein, heat shock 70 kDa protein 1A (*HSPA1A*), is overexpressed under zinc exposure. Those 25 proteins (Table 1) were classified into 35% catalytic enzymes, 13% metal binding proteins, and 11% molecular chaperones according to their molecular functions by literature review and UniProt (Supplementary Figure 3a). PANTHER-based classification showed 29% catalytic enzyme and 21% cytoskeletal proteins (Supplementary Figure 3b). The identified proteins are found to be localised in the cytoplasm (48%), nucleus (22%), and cytoskeleton (13%) (Supplementary Figure 3c).

### 3.4. Differentially Expressed Proteins in MCF10A Breast Normal Epithelial Cells with Exogenous Zinc Exposure Compared to MCF10A Cells without Zinc Exposure

MCF10A normal breast epithelial cells showed overexpression of 7 protein spots (Figure 2(b)) under exogenous zinc exposure for 120 min (*T*_120_) compared to without zinc exposure (*T*_0_). D-3-Phosphoglycerate dehydrogenase (*PHGDH*), elongation factor Tu (*TUFM*), ATP-dependent RNA helicase DDX1 (*DDX1*), inosine-5′-monophosphate dehydrogenase (*IMPDH*), plastin-3 (*PLS3*), radixin (*RDX*), and torsin-1A-interacting protein 1 (*TOR1AIP1*) were related to catalytic activity for cell metabolism and proliferation (Table 1). The classification of these proteins is described in Supplementary Figures 4a–4c.

### 3.5. Differentially Expressed Proteins in PC3 Prostate Cancer Cells against RWPE-1 Normal Prostate Epithelial Cells without Exogenous Zinc Exposure

**Figure 3:**
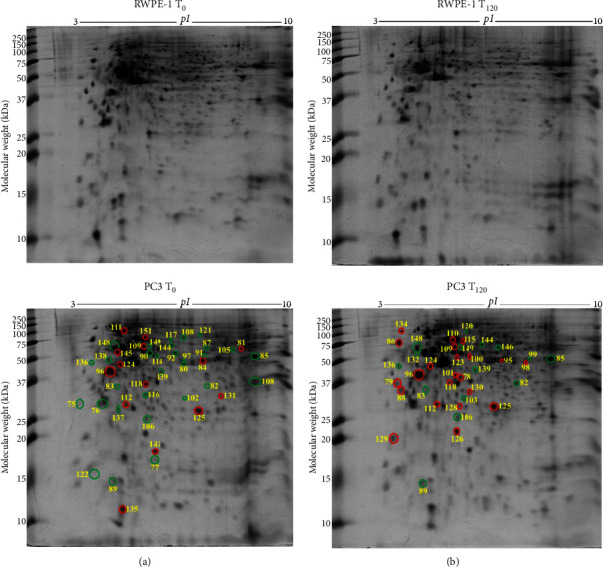
Differentially expressed protein spots in 2-DE gels by comparisons of PC3 *T*_0_ vs RWPE-1 *T*_0_ and PC3 *T*_120_ vs RWPE-1 *T*_120_. (a) Representative 2-DE gel images (in the left panel) of prostate normal RWPE-1 cells without zinc exposure (RWPE-1 *T*_0_) and prostate cancer PC3 cells without zinc exposure (PC3 *T*_0_). (b) Representative 2-DE gel images (in the right panel) of prostate normal RWPE-1 cells with exogenous zinc exposure for 120 min (RWPE-1 *T*_120_) and prostate cancer PC3 cells with exogenous zinc exposure for 120 min (PC3 *T*_120_). Each protein extract (100 *μ*g) was resolved based on isoelectric point (*pI*) and molecular weight (MW). The differentially expressed protein spots are shown with red circles denoting upregulation and green circles denoting downregulation.

PC3 prostate cancer cells showed 30 downregulated (green circle) and 14 upregulated (red circle) protein spots (Figure 3(a)) compared to RWPE-1 prostate normal epithelial cells without zinc exposure (*T*_0_). The abundance of tumour suppressor proteins, such as 14-3-3 protein *σ* (*SFN*), latexin (*LXN*), glutathione S-transferase P (*GSTP1*), Rho GDP-dissociation inhibitor 1 (*ARHGDIA*), serpin B5 (*SERPINB5*), and glycine tRNA ligase (*GARS1*), was reduced in PC3 cells compared to RWPE-1 at *T*_0_ (Table 2). Also reduced are the calcium-binding proteins annexin A1 (*ANXA1*) and annexin A5 (*ANXA5*), mitochondrial ATP synthase subunit *α* (*ATP5F1A*), ATP-dependent RNA helicase DDX39A (*DDX39A*), RNA helicase (*DDX48*), dihydrolipoamide S-succinyltransferase (*DLST*), exosome complex component MTR3 (*EXOSC6*), T-complex protein 1 subunit *α* (*TCP1*), and ubiquitin carboxyl-terminal hydrolase (*USP14*) (Table 2). The upregulated proteins, such as protein S100A6 (*S100A6*), aldehyde dehydrogenase 1 family member A3 isoform (*ALDH1A3*), 26S proteasome non-ATPase regulatory subunit 11 (*PSMD11*), elongation factor 1 *δ* (*EEF1D*), 60 kDa heat shock protein mitochondrial (*HSPD1*), heat shock protein 90 kDa *α* (cytosolic) class B member 1 isoform (*HSP90AB1*), heat shock protein *β* 1 (*HSPB1*), L-lactate dehydrogenase B chain (*LDHB*), peroxiredoxin 6 (*PRDX6*), proteasome subunit *α* type 1 (*PSMA1*), superoxide dismutase (Cu-Zn) (*SOD1*), and acetyltransferase component of pyruvate dehydrogenase complex (*DLAT*), are related to cancer cell proliferation, growth, and invasion (Table 2).

Based on molecular functions, the 44 proteins belong to three prominent classes including catalytic enzymes (34%), molecular chaperones (19%), and metal ion binding proteins (13%) (Supplementary Figure 5a). By using PANTHER database, the 44 proteins are classified into different groups including 29% catalytic enzymes and 14% translational proteins (Supplementary Figure 5b). The proteins apparently localise in the cytoplasm (44%), nucleus (23%), and mitochondrion (12%) (Supplementary Figure 5c).

### 3.6. Differentially Expressed Proteins in PC3 Prostate Cancer Cells Compared to RWPE-1 Normal Prostate Epithelial Cells with Exogenous Zinc Exposure

PC3 prostate cancer cells under zinc exposure for 120 min (*T*_120_) showed 15 downregulated (green circle) and 22 upregulated (red circle) protein spots compared to RWPE-1 cells (Figure 3(b)). Calcium binding proteins annexin A1 (*ANXA1*) and annexin A5 (*ANXA5*) were suppressed and associated with tumorigenesis (Table 2). The suppressed proteins, including mitochondrial ATP synthase subunit *α* (*ATP5F1A*), cytochrome c oxidase subunit 5A mitochondrial (*COX5A*), ethanolamine-phosphate cytidylyltransferase (*PCYT2*), F-actin-capping protein subunit beta (*CAPZB*), glutathione S-transferase P (*GSTP1*), MAD1 mitotic arrest deficient-like 1 (*MAD1L1*), serpin B5 (*SERPINB5*), and ubiquitin carboxyl-terminal hydrolase (*USP14*), and the overexpressed proteins such as L-lactate dehydrogenase B chain (*LDHB*), prostaglandin E synthase 3 (*PTGES3*), and protein kinase C substrate 80K-H isoform (*PRKCSH*) are related to cell proliferation and apoptosis (Table 2). Tumour suppressor protein NDRG1 (*NDRG1*) and prohibitin (*PHB*) were increased in PC3 cells under zinc exposure. The overexpressed proteins, such as 60S acidic ribosomal protein P0 (*RPLP1*), calreticulin (*CALR*), elongation factor 1*δ* (*EEF1D*), elongation factor Tu (*TUFM*), eukaryotic translation initiation factor 3 subunit E (*EIF3E*), and eukaryotic translation initiation factor 3 subunit I (*EIF3I*), are associated with protein translation (Table 2). Molecular chaperones including 60 kDa heat shock protein mitochondrial (*HSPD1*), heat shock 70 kDa protein 1B (*HSPA1B*), and heat shock protein *β*1 (*HSPB1*) were overexpressed (Table 2). Antioxidant proteins peroxiredoxin 6 (*PRDX6*) and peroxiredoxin 2 (*PRDX2*) were also upregulated (Table 2). The 37 proteins are categorised into three key groups including catalytic enzymes (32%), molecular chaperones (19%), and protein synthesis (12%) based on literature and UniProt database (Supplementary Figure 6a). By using PANTHER database, they are classified into catalytic enzymes (34%), molecular chaperones (16%), and translation proteins (16%) (Supplementary Figure 6b), which is in agreement with the categorisation according to molecular function. The identified proteins localise mainly in the cytoplasm (44%), nucleus (20%), and mitochondrion (11%) (Supplementary Figure 6c).

### 3.7. Differentially Expressed Proteins in PC3 Prostate Cancer Cells with Exogenous Zinc Exposure Compared to PC3 Cells without Zinc Exposure

**Figure 4:**
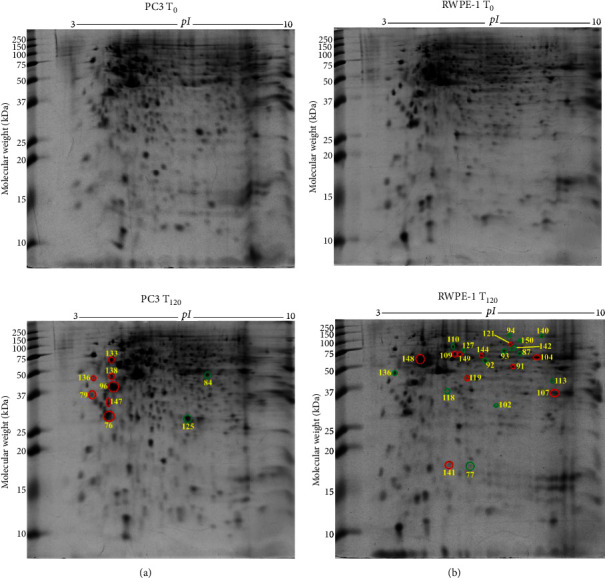
Differentially expressed protein spots in 2-DE gels by comparisons of PC3 *T*_120_ vs PC3 *T*_0_ and RWPE-1 *T*_120_ vs RWPE-1 *T*_0_. (a) Representative 2-DE gel images (in the left panel) of prostate cancer PC3 cells without zinc exposure (PC3 *T*_0_) and with exogenous zinc exposure for 120 min (PC3 *T*_120_). (b) Representative 2-DE gel images (in the right panel) of prostate normal RWPE-1 cells without zinc exposure (RWPE-1 *T*_0_) and with exogenous zinc exposure for 120 min (RWPE-1 *T*_120_). Each protein extract (100 *μ*g) was resolved based on isoelectric point (*pI*) and molecular weight (MW). The differentially expressed protein spots are shown with red circles denoting upregulation and green circles denoting downregulation.

PC3 cells demonstrated 2 suppressed and 7 overexpressed protein spots under exogenous zinc exposure for 120 min (*T*_120_) compared to PC3 cells without zinc exposure (*T*_0_) (Figure 4(a)). Tumour suppressor 14-3-3 protein *θ* (*YWHAQ*) and translational proteins such as 60S acidic ribosomal protein P0 (*RPLP1*), elongation factor 1*δ* (*EEF1D*), and 40S ribosomal protein SA (*RPSA*) were overexpressed (Table 2). Overexpressed tropomyosin 3 isoform 2 (*TPM3*) is related to cancer progression and metastasis (Table 2). Protein disulfide-isomerase (*P4HB*) was also upregulated which serves as molecular chaperone. Peroxiredoxin 6 (*PRDX6*) and 26S proteasome non-ATPase regulatory subunit 11 (*PSMD11*) were downregulated under zinc exposure in PC3 cells (Table 2). The 9 identified proteins showed two prominent molecular functional groups, catalytic enzymes (30%) and protein synthesis (20%) (Supplementary Figure 7a). PANTHER database analysis revealed 38% translational protein class (Supplementary Figure 7b). The identified proteins localise predominantly in the cytoplasm (50%), nucleus (25%), and endoplasmic reticulum (13%) (Supplementary Figure 7c).

### 3.8. Differentially Expressed Proteins in RWPE-1 Prostate Normal Epithelial Cells with Exogenous Zinc Exposure Compared to RWPE-1 Cells without Zinc Exposure

In RWPE-1 cell, 14 suppressed (green circle) and 10 overexpressed (red circle) protein spots were identified following exogenous zinc exposure for 120 min (*T*_120_) compared to RWPE-1 cells without zinc exposure (*T*_0_) (Figure 4(b)). The reduced proteins, such as chaperonin containing TCP1 subunit 6A (*CCT6A*), dihydropyrimidinase-related protein 2 (*DPYSL2*), dihydrolipoamide S-succinyltransferase (*DLST*), succinate dehydrogenase (ubiquinone) flavoprotein subunit mitochondrial (*SDHA*), aspartate aminotransferase (*GOT1*), and L-lactate dehydrogenase B chain (*LDHB*), are related to cellular metabolism and proliferation (Table 2). Upregulated proteins, such as glucose-6-phosphate 1-dehydrogenase (*G6PD*), histone H4 (*HIST1H4J*), tubulin *α*1A chain (*TUBA1A*), L-lactate dehydrogenase (*LDHA*), stathmin (*STMN1*), and ubiquitin carboxyl-terminal hydrolase (*USP14*), are also associated with cell metabolism as well as growth (Table 2). The proteins involved in protein folding such as 60 kDa heat shock protein mitochondrial (*HSPD1*) and T-complex protein 1 subunit *α* (*TCP1*) were overexpressed under zinc exposure (Table 2). Based on molecular functions, the 24 proteins were categorised into catalytic enzymes (34%) and molecular chaperones (23%) (Supplementary Figure 8a). Catalytic enzyme (38%) was the prominent protein class according to PANTHER database analysis (Supplementary Figure 8b). The majority proteins localise in the cytoplasm (48%) and nucleus (22%) (Supplementary Figure 8c).

### 3.9. Functional Interactions of the Differentially Expressed Proteins in Breast and Prostate Cells

By STRING functional protein-protein network analysis, both known and predicted functional interactions were revealed for the differentially expressed proteins in both cancerous and normal breast and prostate cells under the experimental conditions with and without zinc exposure (Supplementary Figures 9 and 10). Heat shock protein 90 kDa *α* (cytosolic) class B member 1 isoform (*HSP90AB1*), actin cytoplasmic 1 (*ACTB*), and triosephosphate isomerase (*TPI1*) are prominent in the functional network derived from the comparison of breast cancer cells (MCF-7) and normal breast epithelial cells (MCF10A) without zinc exposure (Supplementary Figure 9a). Triosephosphate isomerase (*TPI1*) displays its prominence again in the functional network of the differentially expressed proteins in MCF-7 *T*_120_ compared to MCF10A *T*_120_ under zinc exposure (Supplementary Figure 9b). The metal ion binding proteins such as annexin A1 (*ANXA1*), annexin A5 (*ANXA*5), protein S100A2 (*S100A2*), and protein S100A13 (*S100A13*) are at the peripheral edge of the protein network of MCF-7 breast cancer cells without zinc exposure (Supplementary Figure 9a) and again in the network of the differentially expressed proteins in MCF-7 with zinc exposure compared to MCF10A *T*_120_ (Supplementary Figure 9a).

Prohibitin (*PHB*) is prominent in the functional network of the differentially expressed proteins in the prostate cancer cells (PC3) with zinc exposure (Supplementary Figure 10b), apart from the heat shock proteins encoded by *HSPD1*, *HSPA1B*, and *HSPB1*. Heat shock protein 90 kDa *α* (cytosolic) class B member 1 isoform (*HSP90AB1*), proteasome subunit *α* type 1 (*PSMA1*), elongation factor *γ* (*EEF1G*), and 40S ribosomal protein SA (*RPSA*) are predominant in the protein network of the differentially expressed proteins in PC3 cells without zinc exposure (Supplementary Figure 10a).

## 4. Discussion

Zinc dyshomeostasis is the hallmark of breast and prostate cancer cells. Numerous studies have focused on the zinc homeostasis of breast cancer cells or prostate cancer cells, although the current work is the first to investigate these two kinds of cancer cells together in tandem. Furthermore, we examined the total proteomic profiles of breast cancer cells vs normal breast epithelial cells and prostate cancer cells vs normal prostate epithelial cells in the presence or absence of zinc exposure. The differentially expressed proteins with or without zinc exposure in breast cells (MCF-7, MCF10A) (Table 1) and prostate cells (PC3, RWPE-1) (Table 2) in this study are the key datasets, which enhances the understanding of the zinc homeostasis in both breast and prostate cancer cells.

### 4.1. The Intrinsic Differences between the Cancer Cells and Their Normal Counterparts (without Zinc Exposure)

First, the analysis without extracellular zinc treatment demonstrates the intrinsic differences between breast cancer cells MCF-7 and the normal breast epithelial cells MCF10A, as well as between prostate cancer cells PC3 and the normal counterpart RWPE-1 cells. The proteomic results demonstrate a key feature of breast and prostate cancer cells, namely, the downregulation of tumour suppressors or antitumour proteins.

The results showed the reduction of tumour suppressor 14-3-3 protein *σ* and *θ* in MCF-7 and PC3 cancer cells compared to the normal counterparts (Tables 1 and 2), which is in agreement with the previous findings [36, 37]. The 14-3-3 proteins, including seven isoforms such as *σ* and *θ*, are associated with cell cycle, signalling, and apoptosis and are usually downregulated for cancer progression [36, 37]. The tumour suppressor protein S100A2 was decreased in MCF-7 cells (Table 1) as previously reported [38, 39]. However, the expression of S100A2 was unchanged in PC3 cells (Table 2), which is consistent with the previous study [40]. Antitumour proteins such as latexin, glutathione S-transferase P, Rho GDP-dissociation inhibitor 1, and serpin B5 were reduced in their expression in PC3 prostate cancer cells (Table 2), in agreement with the previous studies [41–44]. Annexin A1 was found to be downregulated in MCF-7 and PC3 cancer cells (Tables 1 and 2), which is related to breast and prostate cancer development [45–48]. Also, for the first time, we observed a downregulated antitumour protein, glycine tRNA ligase [49], in PC3 prostate cancer cells but not in breast cancer cells. The downregulation of glycine tRNA ligase could play a role in prostate cancer development.

The proteomic results demonstrate another feature of breast and prostate cancer cells, that is, the upregulation of proteins related to cancer growth and metastasis. *α*-Smooth muscle actin (*α*-SMA) and tumour protein D53 (hD53) were overexpressed in MCF-7 cells (Table 1). *α*-SMA serves as the marker of epithelial-to-mesenchymal transition (EMT) for cancer metastasis [50, 51] and hD53 promotes breast cancer cell proliferation and their expressions are correlated [51]. High expression of F-actin-capping protein subunit *β* (*CAPZB*) in the breast cancer cells (Table 1) is linked with *α*-SMA in regulating breast cancer cell growth and motility [52, 53]. Overexpression of antioxidants in cancer cells enhances the cancer cell proliferation, hence cancer growth in patients. Peroxiredoxin 6, an antioxidant protein, promotes cancer cell proliferation in an oxidative stress environment [54, 55]. Thus, overexpressed peroxiredoxin 6 in MCF-7 cancer cells (1.8-fold, Table 1) and PC3 cancer cells (8.5-fold, Table 2) indicates its role in breast and prostate cancer development. This finding also suggests that peroxiredoxin 6 (*PRDX6*) is a potential target for anticancer drug development. Glutathione S-transferase Mu 3 (*GSTM3*) is another antioxidant overexpressed in MCF-7 breast cancer cells (Table 1), while superoxide dismutase (*SOD1*) was overexpressed in PC3 prostate cancer cells (Table 2). D-3-Phosphoglycerate dehydrogenase, a metabolic enzyme, is involved in redox homeostasis [56]. Its overexpression in MCF-7 breast cancer cells (Table 1) indicates that this enzyme is associated with breast cancer development.

In addition, the results in Tables 1 and 2 demonstrate the overexpression of the proteins related to cancer cell growth, invasion, and metastasis, including heat shock protein *β*1 [57, 58], 60 kDa heat shock protein [59], heterogeneous nuclear ribonucleoproteins C1/C2 [60], histone H4 [61], nucleoside diphosphate kinase, protein S100A13 [62], radixin [63], and triosephosphate isomerase [64]. Metabolic proteins including aldehyde dehydrogenase 1 family member A3, L-lactate dehydrogenase B chain, cytochrome b5 type B and elongation factor Tu, and elongation factor 1*δ* were overexpressed in the breast cancer cells (Table 1) and prostate cancer cells (Table 2). Their overexpression could be related to the cancer cell proliferation. Intriguingly, dihydrolipoamide S-succinyl-transferase (E2 component of 2-oxo-glutarate complex) (*DLST*), a metabolic enzyme of Krebs cycle [65], was upregulated in MCF-7 breast cancer cells (Table 1), but downregulated in PC3 prostate cancer cells (Table 2). The reason for such inverse expression of this enzyme is yet to be examined.

### 4.2. The Dynamic Expression of Proteins in Breast and Prostate Cancer Cells in Response to Zinc Exposure

The proteomic datasets were obtained by the comparison between breast cancer cells MCF-7 and the normal breast epithelial cells MCF10A in response to the change of extracellular zinc concentration, as well as the comparison between prostate cancer cells PC3 and the control cells in response to the change of extracellular zinc. The analysis demonstrates that the cancer cells upregulated the proteins which are related to lysosomal activity, antioxidant activity, stress response, cancer growth, cellular structure, and metabolism.

MCF-7 breast cancer cells showed overexpression of cathepsin D in response to zinc exposure (Table 1). Cathepsin D is an aspartic endoproteinase in lysosome and is well known for its roles in angiogenesis, proliferation, and invasion in breast cancer [66, 67]. The change of extracellular zinc should lead to the elevation of cytoplasmic zinc in MCF-7 cells, which might in turn result in higher zinc level in lysosome and hence cathepsin D upregulation. Because zinc enhances cathepsin D activity in lysosome [68], the overexpression of this endoproteinase might be accompanied with increased proteinase activity in zinc-treated MCF-7 cells. Interestingly, peroxiredoxin 6 was overexpressed only in PC3 prostate cancer cells under the zinc exposure, in contrast to its overexpression previously described in both MCF-7 and PC3 cells without zinc exposure. Additionally, peroxiredoxin 2 was also overexpressed in PC3 cells under zinc exposure. The findings demonstrate that peroxiredoxin 6 is related to the cancer development and stress response while peroxiredoxin 2 is likely more relevant to stress response. Antioxidant proteins, including glutathione S-transferase Mu 3 and mitochondrial NADH dehydrogenase (ubiquinone) iron-sulfur protein 3, were overexpressed in breast cancer cells under zinc exposure (Table 1). A previous study showed that glutathione S-transferase Mu 3 expression has a positive relationship with zinc [69]. The molecular chaperones such as mitochondrial 60 kDa heat shock protein, heat shock 70 kDa protein 1B, and heat shock protein *β*1 were overexpressed in PC3 cancer cells upon zinc exposure (Table 2), which is likely a part of stress response for the prostate cancer cells.

Zinc enhances breast cancer growth. This is evidently supported by the increased intracellular zinc level in breast cancer cells compared to the normal breast epithelial cells [11, 70, 71]. The proteomic dataset showed the elevated expression of tumour protein D53 (hD53 encoded by *TPD52L1*) and tumour protein D54 (hD54 encoded by *TPD52L2*) of MCF-7 breast cancer cells in response to the change of extracellular zinc (Table 1), which explains to some extent why zinc promotes breast cancer growth. This finding also suggests that hD53 and hD54 are potential targets for anticancer drug development against breast cancers.

Intriguingly, the change of extracellular zinc resulted in overexpression of prohibitin (*PHB*) in prostate cancer cells (PC3) (Table 2). Prohibitin can act as a tumour suppressor in prostate cancers [72]. As is known, the intracellular zinc level in prostate cancer cells is lower than the normal counterparts [5, 71]. The variation of extracellular zinc should lead to the increased level of zinc inside the PC3 cancer cells, which is detrimental to the prostate cancer cells. The overexpression of prohibitin might partly explain the cytotoxicity of excess zinc for the prostate cancer cells. Moreover, the reduction of metabolic enzymes including D-3-phosphoglycerate dehydrogenase, adenylosuccinate lyase, inosine-5′-monophosphate dehydrogenase, and translational elongation factor Tu under zinc exposure (Table 1) might be relevant to the decreased cell viability in MCF-7 breast cancer cells under zinc exposure [10], but the expression of these metabolic enzymes is not changed in PC3 prostate cancer cells.

Further proteomic analysis was also done by comparing breast cancer cells MCF-7 with and without zinc treatment, as well as comparing the prostate cancer cells PC3 with and without zinc treatment. Firstly, MCF-7 breast cancer cells exhibited 25 differentially expressed proteins (Table 1) under zinc exposure compared to without zinc exposure (*T*_0_), while PC3 prostate cancer cells showed only 9 differentially expressed proteins (Table 2). This very fact demonstrates that breast cancer cells are more capable responders to the variation of extracellular zinc levels. Their molecular network of zinc homeostasis might be more sophisticated than the one in prostate cancer cells.

The findings demonstrate that zinc upregulates the proteins related to breast cancer growth and metastasis. Zinc exposure upregulated actinin *α*1 and annexin A5 in MCF-7 cells (Table 1). The cytokinetic protein actinin *α*1 is shown to promote tumorigenesis and epithelial-to-mesenchymal transition (EMT) in cancer via AKT/GSK3*β*/*β* catenin signalling pathways [73]. Among 12 annexin A isoforms (annexin A1-11 and annexin A13), annexin A5 in particular has unphosphorylated short N-terminus which enables this protein to exhibit a wide range of functions such as signalling, cancer cell growth, and invasion [74]. The overexpression of both inorganic pyrophosphatase (*PPA1*) and tubulin *α*1c (*TUBA1C*) in response to the variation of exogenous zinc in MCF-7 cells (Table 1) suggests that high intracellular zinc promotes the metabolic activity of breast cancer cells, since inorganic pyrophosphatase is involved in cell metabolism, and tubulin *α*1c promotes glycolysis in breast cancer [75–77]. In addition, current finding demonstrates that heat shock 70 kDa protein was overexpressed in MCF-7 cells (Table 1), correlating well with its overexpression at the gene level [78].

### 4.3. Interactions of the Differentially Expressed Proteins in Cancer Cells

Human triosephosphate isomerase (*TPI1*) is a key glycolytic enzyme, and glycolysis is accelerated in cancer cells [79]. The prominence of triosephosphate isomerase in breast cancer cells (MCF-7) with and without zinc exposure (Supplementary Figures 9a and 9b) demonstrates that it is potentially associated with breast cancer development. The marked upregulation of triosephosphate isomerase in MCF-7 cells without zinc exposure (*T*_0_) and with zinc exposure (*T*_120_) compared to the normal counterparts (Table 1) reflects both its intrinsic expression in the breast cancers and dynamic zinc-responsiveness upon zinc exposure. Triosephosphate isomerase was found to be involved in PI3K/AKT/mTOR signalling pathway and hence breast cancer development [64], which supports the significance of the finding for triosephosphate isomerase in this study. Therefore, it is potentially a druggable target, and it is indeed under investigation for anticancer drug development [80]. The metal-binding proteins, S100A2 and S100A13, belong to S100 protein family, which were first identified by Moore in 1965 [81, 82]. There are 18 members of S100A (S100A1–S100A18). Protein S100A13 is present in the functional networks of MCF-7 with and without zinc exposure (Supplementary Figures 9a and 9b), and it was highly overexpressed both intrinsically in MCF-7 without zinc exposure and responsively to zinc exposure in MCF-7 cells. The findings suggest that S100A13 is involved in zinc homeostasis of breast cancer cells. Prohibitin (*PHB*) is a worthwhile target for future investigations according to its overexpression in the prostate cancer cells (PC3) at *T*_120_ zinc exposure compared to the normal counterparts (Table 2) as well as its prominence in the functional protein network of PC3 *T*_120_ vs RWPE-1 *T*_120_ (Supplementary Figure 10b). Prohibitin is a pleiotropic chaperone/scaffold tumour suppressor protein implicated in the regulation of cell proliferation and apoptosis [83]. This study, for the first time, demonstrated that it is also a zinc-responsive protein in the prostate cancer cells.

## 5. Conclusion

The systematic approach of high-resolution top-down proteomics was carried out simultaneously, for the first time, on the cancerous breast and prostate cells (MCF-7, PC3) and the normal breast and prostate cells (MCF10A, RWPE-1). The datasets revealed the intrinsic differences in the proteomes of cancer cells (MCF-7 and PC3) and their normal counterparts without zinc treatment, such as the downregulation of antitumour proteins (14-3-3 protein *σ*, protein S100A2, latexin, and annexin A1) and the upregulation of tumour protein (hD53), antioxidants (peroxiredoxin 6 and superoxide dismutase), and metabolic enzymes (dihydrolipoamide S-succinyltransferase and aldehyde dehydrogenase 1) in both breast and prostate cancer cells. The zinc-responsive proteomes were then unravelled by their dynamic expressions prodded by the change of extracellular zinc, particularly observed were the increased expressions of tumour proteins (hD53, hD54) and triosephosphate isomerase in breast cancer cells. As the cytoplasmic zinc level is elevated in breast cancer cells, the overexpression of those zinc-responsive proteins could be involved in breast cancer development. Moreover, the upregulation of metal binding protein S100A13 likely plays a role in zinc homeostasis of breast cancer cells. The overexpression of the tumour suppressor prohibitin (*PHB*) in prostate cancer cells (PC3) in response to the change of extracellular zinc provides an explanation for the inhibitory effect of zinc in prostate cancer development. The upregulation of antioxidants in both kinds of cancer cells under zinc exposure, such as peroxiredoxin 6, would benefit cancer cell growth in response to the change of environmental conditions. Overall, the findings here uncovered significant molecular targets for anticancer drug development and enhanced our knowledge as well as understanding of the role of zinc in breast and prostate cancer cells.

## Figures and Tables

**Table 1:** Identified proteins in breast cancer cells (MCF-7) and normal breast epithelial cells (MCF10A) with or without exogenous zinc exposure.

Spot ID	Identified proteins	Gene ID	Protein accession	Theoretical MW (kDa)/*pI*	Observed MW (KDa)/*pI*	PLGS score	Matched peptides	Sequence coverage (%)	(Fold change/*p* value)				Molecular functions
									MCF-7 *T*_0_/MCF10A *T*_0_	MCF-7 *T*_120_/MCF10A *T*_120_	MCF-7 T_120_/MCF-7 *T*_0_	MCF10A *T*_120_/MFC10A *T*_0_	
1	14-3-3 protein *σ*	*SFN*	P31947	27.8/4.5	27.9/4.5	2586	24	44	(0.5/0.02) ↓				I
2	14-3-3 protein *θ*	*YWHAQ*	P27348	27.8/4.5	29.0/4.7	10806	14	43	(0.4/0.003) ↓	(0.3/0.0001) ↓			II
3	26S proteasome non-ATPase regulatory subunit 4	*PSMD4*	Q5VWC4	41.1/4.5	62.0/4.6	1085	22	23			(0.2/0.003) ↓		III
4	39S ribosomal protein L12 mitochondrial	*MRPL12*	P52815	21.3/9.2	17.7/5.3	4066	8	30	(0.7/0.03) ↓				IV
5	60S acidic ribosomal protein P0	*RPLP0*	A0A024RBS2	34.3/5.6	42.0/5.6	588	6	16		(0.3/0.002) ↓			V
6	*α*-Smooth muscle actin 2	*ACTA2*	D2JYH4	42.0/5.1	44.4/5.2	6831	22	28	(6.8/0.0003) ↑		(0.3/0.003) ↓		VI
7	Actin cytoplasmic 1	*ACTB*	P60709	41.7/5.1	59.6/5.3	17835	27	60	(0.3/0.0004) ↓				VII
8	Actinin *α*1 isoform CRA a	*ACTN1*	A0A024R694	103.0/5.1	156.6/5.5	35257	64	45			(1.9/0.02) ↑		VIII
9	Adenosylhomocysteinase	*AHCY*	A0A384MTQ3	47.7/5.9	50.1/6.0	911	24	19			(0.5/0.04) ↓		III
10	Annexin	*ANXA8L1*	A0A075B752	40.7/5.6	35.2/5.6	623	8	17	(0.1/0.04) ↓				VIII
11	Annexin A1	*ANXA1*	P04083	38.7/6.6	36.0/6.6	21563	27	62	(0.2/0.03) ↓				VIII
12	Annexin A5	*ANXA5*	P08758	35.9/4.7	29.0/5.0	3292	8	20	(0.5/0.03) ↓		(4.4/0.02) ↑		VIII
13	Calmodulin-1	*CALM1*	P0DP23	17.0/3.9	13.9/3.3	1613	6	41			(0.2/0.006) ↓		VIII, III
14	Cathepsin D	*CTSD*	A0A1B0GW44	43.7/6.1	30.2/5.4	1908	11	19		(8.4/0.0001) ↑	(3.3/0.007) ↑		III
15	Cytochrome b5 type B	*CYB5B*	O43169	16.3/4.7	18.2/3.7	867	3	17	(4.2/0.0003) ↑				VIII
16	D-3-Phosphoglycerate dehydrogenase	*PHGDH*	A0A286YF22	55.9/6.3	69.2/6.7	4302	8	16	(2.0/0.02) ↑	(0.8/0.04) ↓		(1.4/0.02) ↑	III
17	Dihydrolipoamide S-succinyltransferase (E2 component of 2-oxo-glutarate complex) isoform CRA a	*DLST*	A0A024R6C9	48.7/9.3	62.0/6.0	1601	4	10	(2.6/0.01) ↑				III
18	Dynactin 2 (P50) isoform CRA c	*DCTN2*	A0A384MDU9	44.2/4.9	59.2/5.2	6320	21	52		(0.4/0.03) ↓	(0.7/0.04) ↓		VII
19	Elongation factor 1 *δ*	*EEF1D*	A0A087X1X7	69.2/6.8	42.0/4.3	52531	21	25			(0.7/0.001) ↓		V
20	Elongation factor Tu	*TUFM*	A0A384ME17	49.8/7.4	50.1/7.0	2644	49	36	(5.2/0.01) ↑	(0.5/0.003) ↓	(0.5/0.01) ↓	(1.4/0.02) ↑	V
21	Exosome complex component MTR3	*EXOSC6*	Q5RKV6	28.2/6.0	32.4/5.9	350	4	17		(0.5/0.04) ↓			IV
22	F-Actin-capping protein subunit *β*	*CAPZB*	A0A384MR50	30.6/5.6	28.5/5.7	1812	10	26	(3.0/0.0003) ↑		(2.4/0.03) ↑		VI
23	ATP-dependent RNA helicase DDX1	*DDX1*	A0A087X2G1	73.9/7.6	136.2/8.1	298	19	12				(1.4/0.04) ↑	III
24	Adenylosuccinate lyase	*ADSL*	A0A1B0GTJ7	54.4/6.7	67.2/7.6	584	4	8	(0.3/0.002) ↓	(0.4/0.005) ↓			III
25	FUBP1	*FUBP1*	A0A1Z1G4M2	67.6/7.9	89.5/8.2	372	5	9	(2.1/0.02) ↑				II
26	Peptidyl-prolyl cis-trans isomerase FKBP4	*FKBP4*	Q02790	51.8/5.2	62.0/5.6	32827	47	65		(1.9/0.01) ↑	(2.5/0.003) ↑		IX
27	Glutathione S-transferase Mu 3	*GSTM3*	P21266	26.5/5.2	26.9/5.4	8436	20	54	(3.0/0.04) ↑	(5.0/0.005) ↑			III
28	Glutathione S-transferase P	*GSTP1*	P09211	23.3/5.3	24.6/5.6	12769	31	66		(0.2/0.002) ↓			III
29	Glutathione synthetase	*GSS*	P48637	52.4/5.6	62.0/5.6	11269	26	57	(1.5/0.04) ↑				III
30	Inorganic pyrophosphatase	*PPA1*	Q15181	32.6/5.4	37.8/5.5	957	4	11			(2.5/0.03) ↑		III
31	Heat shock protein 90 kDa alpha (cytosolic) class B member 1 isoform CRA a	*HSP90AB1*	A0A024RD80	83.2/4.8	143.0/5.4	5834	28	33	(0.5/0.0006) ↓				IX
32	Heat shock protein *β* 1	*HSPB1*	P04792	22.8/6.0	26.0/5.9	46679	22	78	(3.5/0.002) ↑		(0.2/0.002) ↓		IX
33	Pyruvate dehydrogenase E1 component subunit *α*	*PDHA1*	A0A024RBX9	43.3/8.0	50.1/6.5	373	3	6	(1.7/0.04) ↑				III
34	Heterogeneous nuclear ribonucleoproteins C1/C2	*HNRNPC*	B2R5W2	31.9/4.9	40.8/5.0	7292	24	43	(8.0/0.0002) ↑		(0.5/0.009) ↓		X, IV
35	High mobility group protein B1	*HMGB1*	A0A024RDR0	24.9/5.5	23.5/6.3	3179	8	26	(1.9/0.03) ↑				X
36	Histidine-tRNA ligase cytoplasmic	*HARS1*	P12081	57.4/5.6	62.0/5.8	3224	15	25			(1.5/0.04) ↑		III
37	Histone H4	*HIST1H4J*	B2R4R0	11.4/11.8	18.2/5.7	897	4	36	(2.5/0.007) ↑	(1.9/0.001) ↑			VII, X
38	Heat shock 70 kDa protein 1A	*HSPA1A*	A0A1U9X7W4	70.0/5.3	89.5/5.5	29565	38	49			(1.7/0.02) ↑		IX
39	Inosine-5′-monophosphate dehydrogenase	*IMPDH*	A0A384N6C2	55.8/6.5	69.2/7.0	3921	8	14		(0.6/0.04) ↓		(1.5/0.005) ↑	III
40	L-Lactate dehydrogenase B chain	*LDHB*	P07195	36.6/5.6	38.9/5.7	6282	11	28		(0.6/0.004) ↓			III
41	C-1-Tetrahydrofolate synthase, cytoplasmic	*MTHFD1*	A0A024R652	101.5/6.8	156.6/7.8	342	18	18	(0.5/0.04) ↓				XI, V
42	Myosin regulatory light chain 12A	*MYL12A*	J3QRS3	20.4/4.4	17.3/4.8	1142	5	29	(0.4/0.0003) ↓				VIII
43	NADH dehydrogenase (ubiquinone) iron-sulfur protein 3 mitochondrial	*NDUFS3*	O75489	30.2/7.4	29.0/5.7	1561	5	20		(2.5/0.0004) ↑			III
44	Nucleoside diphosphate kinase	*NME*	A0A384MTW7	17.1/5.8	19.1/5.8	3957	6	30	(2.8/0.01) ↑	(1.7/0.03) ↑			III
45	Perilipin	*PLIN*	A0A140VJN8	46.9/5.1	55.1/5.1	3760	11	32		(0.1/0.005) ↓			XII
46	Plastin 3	*PLS3*	P13797	70.8/5.3	92.2/5.6	4429	20	31				(1.8/0.04) ↑	VIII
47	Actin *γ*	*ACTG2*	P63267	41.9/5.2	47.1/5.1	11163	11	30		(5.2/0.001) ↑			VII
48	Proliferating cell nuclear antigen	*PCNA*	P12004	28.8/4.4	36.0/4.7	3412	13	44	(1.5/0.004) ↑	(0.6/0.02) ↓			X
49	Peroxiredoxin 6	*PRDX6*	A0A024R938	25.0/6.0	26.0/6.3	2521	26	34	(1.9/0.04) ↑				III
50	Proteasome subunit *β* type 6	*PSMB6*	P28072	25.3/4.6	21.4/4.9	1361	5	21		(1.4/0.04) ↑	(0.3/0.03) ↓		III
51	Protein S100A2	*S100A2*	P29034	11.0/4.5	12.0/4.5	2026	3	18	(0.04/0.004) ↓	(0.08/0.01) ↓	(0.4/0.02) ↓		VIII
52	Cytochrome c oxidase subunit 6B1	*COX6B1*	P14854	10.2/6.9	12.4/5.9	833	8	28	(1.9/0.02) ↑		(0.7/0.04) ↓		XII
53	Protein S100A13	*S100A13*	Q99584	11.5/5.8	13.1/5.7	9088	17	60	(4.1/0.001) ↑	(4.0/0.0002) ↑			VIII
54	Protein SET	*SET*	Q01105	33.5/4.0	50.1/4.4	8811	16	34	(0.2/0.0009) ↓	(0.2/0.0001) ↓			IX, X
55	Radixin	*RDX*	B0YJ88	68.5/6.0	110.1/6.3	5828	28	36	(1.3/0.02) ↑	(0.3/0.0004) ↓		(1.6/0.004) ↑	VI
56	Rho GDP-dissociation inhibitor 1	*ARHGDIA*	P52565	23.2/4.8	26.8/5.1	1996	8	28		(0.6/0.04) ↓			III
57	40S ribosomal protein SA	*RPSA*	A0A024R2L6	32.8/4.6	47.1/5.1	2510	8	16		(4.4/0.0005) ↑			VII
58	60S acidic ribosomal protein P2	*RPLP2*	A0A024RCA7	11.7/4.2	15.9/4.3	8497	11	77	(0.7/0.004) ↓				V
59	Serine/threonine-protein kinase PAK 2	*PAK2*	Q13177	58.0/5.6	73.4/5.8	2239	17	28			(0.5/0.03) ↓		III
60	Serpin B5	*SERPINB5*	A0A024R2B6	42.1/5.6	45.7/5.8	2466	15	38		(0.2/0.0007) ↓			III
61	START domain-containing 10 isoform CRA a	*STARD10*	A0A024R5L8	33.0/6.7	39.8/6.0	1134	5	20		(3.1/0.002) ↑			XIII
62	Stathmin	*STMN1*	P16949	17.3/5.7	16.5/5.7	8524	12	53			(0.4/0.002) ↓		VII
63	SUMO-1 activating enzyme subunit 1 isoform CRA b	*SAE1*	A0A024R0R4	38.4/5.0	42.0/5.4	3930	11	28	(0.1/0.03) ↓				III
64	T-Complex protein 1 subunit *α*	*TCP1*	P17987	60.3/5.7	75.5/5.8	4508	17	30		(0.4/0.008) ↓			IX
65	T-Complex protein 1 subunit *γ*	*CCT3*	B3KX11	57.9/6.5	89.5/6.1	1050	17	23	(0.8/0.02) ↓				IX
66	T-Complex protein 1 subunit *θ*	*CCT8*	P50990	59.6/5.3	73.4/5.6	1289	15	28	(0.2/0.03) ↓				IX
67	Thioredoxin	*TXN*	H9ZYJ2	11.7/4.6	14.5/5.1	1389	15	34	(1.4/0.0004) ↑				III
68	Torsin-1A-interacting protein 1	*TOR1AIP1*	A0A0A0MSK5	52.4/6.6	75.5/6.1	1086	9	21		(0.2/0.001) ↓		(2.3/0.0005) ↑	III
69	Transitional endoplasmic reticulum ATPase	*VCP*	P55072	89.3/5.0	132.3/5.4	2552	27	28			(0.6/0.01) ↓		V, III
70	Triosephosphate isomerase	*TPI1*	P60174	30.8/5.6	25.5/7.7	4224	8	32	(3.0/0.002) ↑	(3.8/0.03) ↑			III
71	Tubulin alpha-1C chain	*TUBA1C*	Q9BQE3	50.1/4.8	14.8/5.3	1665	4	12	(0.2/0.0001) ↓	(0.2/00) ↓	(3.1/0.03) ↑		VII
72	Tumour protein D53	*TPD52L1*	E9PNK6	18.7/5.5	19.1/5.6	1601	3	9	(4.8/0.0005) ↑	(13.6/0.001) ↑			II
73	Tumour protein D54	*TPD52L2*	A0A087WYR3	23.8/6.1	27.9/5.2	3042	11	38		(2.5/0.04) ↑			II
74	Vesicle amine transport protein 1	*VAT1*	A0A024R1Z6	41.9/5.9	57.5/6.0	5485	12	21			(0.7/0.04) ↓		III

*Note*. MW stands for molecular weight, kDa for kilo Dalton, *pI* for isoelectric point, PLGS for ProteinLynx Global Server, *T*_0_ for 0 min or without zinc exposure (control), *T*_120_ for 120 min, ↑ for upregulation, ↓ for downregulation, MCF-7 for breast cancer cells, and MCF10A for breast normal epithelial cells. The PLGS score, protein accession, theoretical MW/*pI*, matched peptides, and sequence coverage (%) were obtained using ProteinLynx Global Server (PLGS) software (version 3.0, Waters Corporation, USA) and the UniProt (*Homo sapiens*, human) database. Gene ID was derived from UniProt database. The observed MW and *pI* were calculated according to the protein standards. The fold changes and *p* values were acquired from the quantitative analysis of the gel images (each group *n* = 3) by Delta2D software (version 4.0.8, DECODON Gmbh, Germany). MCF-7 *T*_0_/MCF10A *T*_0_ is the expression fold change of the proteins in MCF-7 cells compared to MCF10A cells without zinc exposure (*T*_0_), MCF-7 *T*_120_/MCF10A *T*_120_ is the expression fold change of the proteins in MCF-7 cells compared to MCF10A cells following the zinc exposure for 120 min (*T*_120_), MCF-7 *T*_120_/MCF-7 *T*_0_ is the expression fold change of the proteins in MCF-7 cells following zinc exposure for *T*_120_ compared to *T*_0_, and MCF10A *T*_120_/MFC10A *T*_0_ is the fold change of the proteins in MCF10A cells following zinc exposure for *T*_120_ compared to *T*_0_. Molecular functions: I, apoptosis; II, signalling; III, catalytic activity; IV, RNA binding; V, protein synthesis; VI, protein binding; VII, structural; VIII, metal ion binding; IX, molecular chaperone; X, DNA binding; XI, DNA synthesis; XII, metabolism; XIII, lipid binding.

**Table 2:** Identified proteins in prostate cancer cells (PC3) and normal prostate epithelial cells (RWPE-1) with or without exogenous zinc exposure.

Spot ID	Identified proteins	Gene ID	Protein accession	Theoretical MW (kDa)/*pI*	Observed MW (kDa)/*pI*	PLGS score	Matched peptides	Sequence coverage (%)	(Fold change/*p* value)				Molecular functions
									PC3 *T*_0_/RWPE-1 *T*_0_	PC3 *T*_120_/RWPE-1 *T*_120_	PC3 *T*_120_/PC3 *T*_0_	RWPE-1 *T*_120_/RWPE-1 *T*_0_	
75	14-3-3 protein *σ*	*SFN*	P31947	27.8/4.5	27.3/3.8	932	3	6	(0.4/0.001) ↓				I
76	14-3-3 protein *θ*	*YWHAQ*	P27348	27.8/4.5	27.3/4.7	15878	15	40	(0.4/0.003) ↓		(1.5/0.0009) ↑		II
77	40S ribosomal protein S18	*RPS18*	P62269	17.7/11.4	17.5/5.7	2004	6	38	(0.5/0.03) ↓			(0.3/0.02) ↓	III, IV
78	Transaldolase	*TALDO1*	A0A140VK56	37.5/6.4	39.1/5.6	423	11	18		(1.7/0.01) ↑			V
79	60S acidic ribosomal protein P0	*RPLP1*	A0A024RBS2	34.3/5.6	42.3/4.1	208	3	7		(1.4/0.01) ↑	(1.5/0.006) ↑		VI
80	Adenosylhomocysteinase	*AHCY*	A0A384MTQ3	47.7/5.9	50.9/5.9	657	20	22	(0.5/0.005) ↓				V
81	Aldehyde dehydrogenase 1 family member A3 isoform CRA a	*ALDH1A3*	A0A024RC95	56.1/7	74.4/7.7	3883	39	30	(1.5/0.02) ↑				V
82	Annexin A1	*ANXA1*	P04083	38.7/6.6	42.6/6.6	24829	27	55	(0.2/0.02) ↓	(0.3/0.04) ↓			VII
83	Annexin A5	*ANXA5*	P08758	35.9/4.7	33.4/5.1	12361	20	50	(0.2/0.0008) ↓	(0.3/0.004) ↓			VII
84	26S proteasome non-ATPase regulatory subunit 11	*PSMD11*	O00231	47.4/6.1	53.9/6.7	1037	18	34	(4.0/0.0009) ↑		(0.3/0.004) ↓		V
85	ATP synthase subunit *α* mitochondrial	*ATP5F1A*	P25705	59.7/9.4	56.3/7.8	26896	25	45	(0.4/0.008) ↓	(0.6/0.04) ↓			III, V
86	Calreticulin	*CALR*	P27797	48.1/4.1	81.3/4.4	9707	32	70		(1.7/0.007) ↑			VIII
87	Chaperonin containing TCP1 subunit 6A (*ζ* 1) isoform CRA a	*CCT6A*	A0A024RDL1	58/6.2	78.0/6.2	1700	21	33	(0.7/0.02) ↓			(0.6/0.003) ↓	VIII
88	Clathrin light chain A	*CLTA*	P09496	27/4.2	35.4/4.2	1196	8	14		(1.8/0.02) ↑			IV
89	Cytochrome c oxidase subunit 5A mitochondrial	*COX5A*	H3BRM5	7.8/5.7	14.2/5.0	1969	4	35	(0.6/0.01) ↓	(0.5/0.03) ↓			VII
90	ATP-dependent RNA helicase DDX39A	*DDX39A*	O00148	49.1/5.3	62.9/5.6	3285	8	14	(0.6/0.004) ↓				V
91	RNA helicase	*DDX48*	A0A024R8W0	46.8/6.3	58.3/6.2	4579	49	38	(0.5/0.02) ↓			(2.9/0.006) ↑	V
92	Dihydrolipoamide S-succinyltransferase (E2 component of 2-oxo-glutarate complex) isoform CRA a	*DLST*	A0A024R6C9	48.7/9.3	62.9/5.8	2675	12	18	(0.2/0.0003) ↓			(0.6/0.03) ↓	V
93	Dihydropyrimidinase-related protein 2	*DPYSL2*	A0A1C7CYX9	73.5/5.9	81.9/6.0	3385	19	29				(0.3/0.001) ↓	V
94	Dopamine receptor interacting protein 4	*DRIP4*	Q4W4Y1	96.0/6.1	158.9/6.1	5782	44	38				(0.3/0.004) ↓	II
95	S-Adenosylmethionine synthase	*MAT2A*	B4DEX8	39.7/5.6	52.2/6.1	1183	16	27		(1.3/0.03) ↑			V
96	Elongation factor 1 *δ*	*EEF1D*	A0A087X1X7	69.2/6.8	40.1/5.0	26412	18	23	(1.6/0.03) ↑	(1.4/0.04) ↑	(1.2/0.03) ↑		VI
97	Elongation factor 1 *γ*	*EEF1G*	P26641	50.1/6.2	54.3/6.0	2159	10	18	(0.4/0.004) ↓				VI
98	Elongation factor Tu	*TUFM*	A0A384ME17	49.8/7.4	50.5/6.7	2726	51	33		(5.1/0.0004) ↑			VI
99	Ethanolamine-phosphate cytidylyltransferase	*PCYT2*	I3L1R7	41.4/7.0	54.3/7.2	803	9	23		(0.1/0.008) ↓			IX
100	Eukaryotic translation initiation factor 3 subunit E	*EIF3E*	B2R806	52.2/5.6	57.9/5.7	1850	11	21		(2.0/0.002) ↑			VI
101	Eukaryotic translation initiation factor 3 subunit I	*EIF3I*	Q13347	36.5/5.3	40.1/5.6	1091	6	17		(3.4/0.0003) ↑			VI
102	Exosome complex component MTR3	*EXOSC6*	Q5RKV6	28.2/6.0	29.5/5.9	795	13	19	(0.3/0.001) ↓			(0.6/0.01) ↓	III
103	F-Actin-capping protein subunit *β*	*CAPZB*	A0A384MR50	30.6/5.6	30.1/5.7	3939	12	27		(0.6/0.006) ↓			X
104	Glucose-6-phosphate 1-dehydrogenase	*G6PD*	A0A384NL00	59.2/6.4	71.2/7.1	8928	24	38				(5.5/0.01) ↑	IX
105	Glutamate dehydrogenase	*GLUD1*	B4DMF5	56.6/6.8	65.4/7.5	4823	19	37	(0.6/0.009) ↓				IX
106	Glutathione S-transferase P	*GSTP1*	P09211	23.3/5.3	24.6/5.6	21246	47	67	(0.3/0.006) ↓	(0.4/0.0002) ↓			V
107	Histone H4	*HIST1H4J*	B2R4R0	11.4/11.8	34.9/9.2	3206	8	62	(0.3/0.02) ↓			(1.5/0.03) ↑	IV
108	Glycine tRNA ligase	*GARS1*	A0A090N8G0	77.5/5.8	116.0/5.9	5889	112	41	(0.3/0.00001) ↓				V
109	60 kDa heat shock protein, mitochondrial	*HSPD1*	A0A024R3W0	61.0/5.6	70.7/5.6	16269	32	48	(1.5/0.02) ↑	(1.9/0.0005) ↑		(2.9/0.01) ↑	VIII
110	Heat shock 70 kDa protein 1B	*HSPA1B*	A0A0G2JIW1	70.1/5.3	90.2/5.6	10234	35	40		(1.5/0.02) ↑		(0.5/0.004) ↓	VIII
111	Heat shock protein 90 kDa alpha (cytosolic) class B member 1 isoform CRA a	*HSP90AB1*	A0A024RD80	83.2/4.8	123.6/5.3	10935	37	41	(1.8/0.002) ↑				VIII
112	Heat shock protein *β* 1	*HSPB1*	P04792	22.8/6.0	27.1/5.4	5134	9	42	(2.1/0.02) ↑	(2.2/0.02) ↑			VIII
113	Aspartate aminotransferase	*GOT1*	A0A140VK69	46.2/6.6	46.4/7.5	682	20	24				(0.40/0.004) ↓	V
114	Histidine tRNA ligase, cytoplasmic	*HARS1*	B4DDD8	48.5/5.0	58.3/5.6	548	5	11	(0.6/0.03) ↓				V
115	Copine 1	*CPNE1*	B0QZ18	59.7/5.6	90.2/5.6	11670	27	26		(1.8/0.004) ↑			V
116	Latexin	*LXN*	Q9BS40	25.7/5.4	29.8/5.6	1946	19	23	(0.8/0.02) ↓				II
117	Leukotriene A (4) hydrolase	*LTA4H*	A0A140VK27	69.2/5.7	90.9/5.8	2315	27	24	(0.2/0.01) ↓				VII
118	L-Lactate dehydrogenase B chain	*LDHB*	A0A5F9ZHM4	37.4/5.8	34.6/5.6	2746	8	23	(2.0/0.01) ↑	(2.2/0.0003) ↑		(0.7/0.03) ↓	V
119	L-Lactate dehydrogenase	*LDHA*	A0A3B3IS95	30.7/6.1	45.0/5.7	3821	14	25				(1.9/0.02) ↑	V
120	MAD1 mitotic arrest deficient-like 1	*MAD1L1*	A4D218	91.7/8.1	134.2/4.8	738	23	26		(0.5/0.04) ↓			II
121	Moesin	*MSN*	P26038	67.8/6.0	102.0/6.1	3846	26	32	(0.7/0.004) ↓			(1.6/0.002) ↑	VIII
122	Myosin light polypeptide 6	*MYL6*	B7Z6Z4	26.7/4.8	14.1/4.7	5045	6	24	(0.3/0.006) ↓				VII
123	Protein NDRG1	*NDRG1*	A0A024R9I3	39.5/6.1	53.9/5.6	8387	11	27		(1.8/0.03) ↑			II
124	NSFL1 cofactor p47	*NSFL1C*	Q9UNZ2	40.6/4.8	47.4/5.2	11230	29	65	(5.0/0.0003) ↑	(2.4/0.004) ↑			VIII
125	Peroxiredoxin 6	*PRDX6*	A0A024R938	25.0/6.0	25.3/6.2	4671	45	52	(8.4/0.0007) ↑	(2.8/0.02) ↑	(0.2/0.03) ↓		V
126	Peroxiredoxin 2	*PRDX2*	P32119	21.9/5.6	20.9/5.6	4964	13	40		(2.4/0.0003) ↑			V
127	Plastin 3	*PLS3*	P13797	69.3/5.5	86.1/5.6	1149	12	17				(0.6/0.01) ↓	VII
128	Prohibitin	*PHB*	A8K401	29.8/5.4	26.6/5.6	3671	17	55		(2.5/0.0001) ↑			XI
129	Prostaglandin E synthase 3	*PTGES3*	A0A087WYT3	19.1/4.2	19.8/4.1	339	11	14		(2.1/0.03) ↑			VIII
130	Proteasome (prosome macropain) activator subunit 3 (PA28 *γ* ki) isoform CRA a	*PSME3*	A0A024R203	30.9/6.3	31.7/5.7	3923	12	36		(1.7/0.04) ↑			I
131	Proteasome subunit *α* type 1	*PSMA1*	P25786	29.5/6.2	29.8/6.8	3657	10	35	(9.5/0.0005) ↑				III
132	Protein DDI1 homolog 2	*DDI2*	Q5TDH0	44.5/4.8	56.3/5.1	320	4	12		(0.2/0.007) ↓			V
133	Protein disulfide-isomerase	*P4HB*	A0A024R8S5	57.1/4.6	67.7/4.9	21868	53	66			(1.4/0.03) ↑		V
134	Protein kinase C substrate 80K-H isoform CRA a (glucosidase 2 subunit beta)	*PRKCSH*	A0A024R7F1	59.3/4.1	133.2/4.5	2052	10	18		(2.1/0.01) ↑			V, VII
135	Protein S100A6	*S100A6*	P06703	10.2/5.2	12.1/5.3	3080	6	33	(1.6/0.039) ↑				VII
136	Protein SET	*SET*	Q01105	33.5/4.0	50.9/4.4	4249	14	33	(0.2/0.0002) ↓	(0.4/0.0001) ↓	(1.7/0.02) ↑	(0.7/0.004) ↑	VIII, XII
137	Rho GDP-dissociation inhibitor 1	*ARHGDIA*	P52565	23.2/4.8	25.5/5.1	4058	13	43	(0.6/0.006) ↓				V
138	40S ribosomal protein SA	*RPSA*	A0A024R2L6	32.8/4.6	52.2/5.0	8860	69	27	(0.5/0.0003) ↓		(1.5/0.006) ↑		IV
139	Serpin B5	*SERPINB5*	A0A024R2B6	42.1/4.9	42.6/5.7	14963	33	63	(0.3/0.002) ↓	(0.4/0.006) ↓			V
140	Staphylococcal nuclease domain-containing protein	*SND1*	A0A140VK49	101.9/6.8	158.9/7.6	4962	70	48				(0.3/0.00009) ↓	V
141	Stathmin	*STMN1*	P16949	17.3/5.7	18.0/5.4	2472	9	45				(5.1/0.03) ↑	IV
142	Succinate dehydrogenase (ubiquinone) flavoprotein subunit mitochondrial	*SDHA*	A0A024QZ30	72.7/7.0	90.9/6.2	2908	15	20				(0.6/0.03) ↓	V
143	Superoxide dismutase (Cu-Zn)	*SOD1*	P00441	15.9/6.7	17.4/5.7	6360	3	18	(2.3/0.004) ↑				VIII
144	T-Complex protein 1 subunit *α*	*TCP1*	P17987	60.3/5.7	74.4/5.8	4205	14	22	(0.6/0.025) ↓	(0.4/0.05) ↓		(4.6/0.02) ↑	VIII
145	Eukaryotic translation initiation factor 3 subunit F	*EIF3F*	B3KSH1	39.1/5.1	54.3/5.1	10070	7	25	(1.4/0.005) ↑				II
146	Torsin-1A-interacting protein 1	*TOR1AIP1*	A0A0A0MSK5	52.4/6.6	71.2/6.1	1483	14	33		(0.2/0.003) ↓			V
147	Tropomyosin 3 isoform 2	*TPM3*	A0A0S2Z4G8	28.7/4.5	32.4/4.9	12626	25	63			(1.5/0.002) ↑		X
148	Tubulin *α* 1A chain	*TUBA1A*	Q71U36	50.1/4.8	68.2/5.1	74078	20	49	(0.5/0.002) ↓	(0.6/0.007) ↓		(1.3/0.02) ↑	IV
149	Ubiquitin carboxyl-terminal hydrolase	*USP14*	A6NJA2	51.1/5.6	78.0/5.6	2543	4	8	(0.5/0.03) ↓	(0.5/0.003) ↓		(3.0/0.004) ↑	V
150	Zyxin	*ZYX*	Q15942	61.2/6.2	116.0/6.2	768	7	16				(0.6/0.02) ↓	VII
151	Acetyltransferase component of pyruvate dehydrogenase complex	*DLAT*	B4DJX1	62.7/5.4	101.2/5.6	1844	24	17	(1.6/0.01) ↑				V

*Note*. MW stands for molecular weight, kDa for kilo Dalton, *pI* for isoelectric point, PLGS for ProteinLynx Global Server, *T*_0_ for 0 min or without zinc exposure (control), *T*_120_ for 120 min, ↑ for upregulation, ↓ for downregulation, PC3 for prostate cancer cells, and RWPE-1 for prostate normal epithelial cells. The PLGS score, protein accession, theoretical MW/*pI*, matched peptides, and sequence coverage (%) were obtained using ProteinLynx Global Server (PLGS) software (version 3.0, Waters Corporation, USA) and the UniProt (*Homo sapiens*, human) database. Gene ID was derived from UniProt database. The observed MW and *pI* were calculated according to the protein standards. The fold changes and *p* values were acquired from the quantitative analysis of the gel images (each group *n* = 3) by Delta2D software (version 4.0.8, DECODON Gmbh, Germany). PC3 *T*_0_/RWPE-1 *T*_0_ is the expression fold change of the proteins in PC3 cells compared to RWPE-1 cells without zinc exposure (*T*_0_), PC3 *T*_120_/RWPE-1 *T*_120_ is the expression fold change of the proteins in PC3 cells compared to RWPE-1 cells following the zinc exposure for 120 min (*T*_120_), PC3 *T*_120_/PC3 *T*_0_ is the expression fold change of the proteins in PC3 cells following zinc exposure for *T*_120_ compared to *T*_0_, and RWPE-1 *T*_120_/RWPE-1 *T*_0_ is the expression fold change of the proteins in RWPE-1 cells following zinc exposure for *T*_120_ compared to *T*_0_. Molecular functions: I, apoptosis; II, signalling; III, RNA binding; IV, structural; V, catalytic activity; VI, protein synthesis; VII, metal ion binding; VIII, molecular chaperone; IX, metabolism; X, protein binding; XI, transcription; XII, DNA binding.

## Data Availability

All data are included in the article and the supplementary file.

## Supplementary Materials

Supplementary Materials The supplementary file includes functional classifications of the identified proteins in MCF-7 breast cancer cells compared to MCF10A breast normal epithelial cells at *T*_0_ (without extracellular zinc exposure) and *T*_120_ under extracellular zinc exposure; functional classifications of the identified proteins in MCF-7 breast cancer cells with extracellular zinc exposure for 120 min (*T*_120_) compared to without zinc exposure (*T*_0_); functional classifications of the identified proteins in MCF10A breast normal epithelial cells with extracellular zinc exposure for 120 min (*T*_120_) compared to without zinc exposure (*T*_0_); functional classifications of the identified proteins in PC3 prostate cancer cells compared to RWPE-1 prostate normal epithelial cells at *T*_0_ (without extracellular zinc exposure) and *T*_120_ under extracellular zinc exposure; functional classifications of the identified proteins in PC3 prostate cancer cells with extracellular zinc exposure for 120 min (*T*_120_) compared to without zinc exposure (*T*_0_); functional classifications of the identified proteins in RWPE-1 prostate normal epithelial cells with extracellular zinc exposure for 120 min (*T*_120_) compared to without zinc exposure (*T*_0_); functional interactions of the differentially expressed proteins in breast cancerous MCF-7 cells and breast normal epithelial MCF10A cells without and with extracellular zinc exposure by the STRING analysis; and functional interactions of the differentially expressed proteins in prostate cancerous PC3 cells and prostate normal epithelial RWPE-1 cells without and with extracellular zinc exposure by the STRING analysis.
