# Supplementary material for: Molecular Insights into the Breast and Prostate Cancer Cells in Response to the Change of Extracellular Zinc
Source: J Oncol. 2024 Jan 12;2024:9925970. doi: 10.1155/2024/9925970 (PMC10798840; doi:10.1155/2024/9925970)
Supplement: Supplementary Materials — The supplementary file includes functional classifications of the identified proteins in MCF-7 breast cancer cells compared to MCF10A breast normal epithelial cells at T0 (without extracellular zinc exposure) and T120 under extracellular zinc exposure; functional classifications of the identified proteins in MCF-7 breast cancer cells with extracellular zinc exposure for 120 min (T120) compared to without zinc exposure (T0); functional classifications of the identified proteins in MCF10A breast normal epithelial cells with extracellular zinc exposure for 120 min (T120) compared to without zinc exposure (T0); functional classifications of the identified proteins in PC3 prostate cancer cells compared to RWPE-1 prostate normal epithelial cells at T0 (without extracellular zinc exposure) and T120 under extracellular zinc exposure; functional classifications of the identified proteins in PC3 prostate cancer cells with extracellular zinc exposure for 120 min (T120) compared to without zinc exposure (T0); functional classifications of the identified proteins in RWPE-1 prostate normal epithelial cells with extracellular zinc exposure for 120 min (T120) compared to without zinc exposure (T0); functional interactions of the differentially expressed proteins in breast cancerous MCF-7 cells and breast normal epithelial MCF10A cells without and with extracellular zinc exposure by the STRING analysis; and functional interactions of the differentially expressed proteins in prostate cancerous PC3 cells and prostate normal epithelial RWPE-1 cells without and with extracellular zinc exposure by the STRING analysis. [file 9925970.f1.docx]

**Supplementary Information**

| **(a) Molecular function** | | **(b) Protein class** | |
| --- | --- | --- | --- |
| 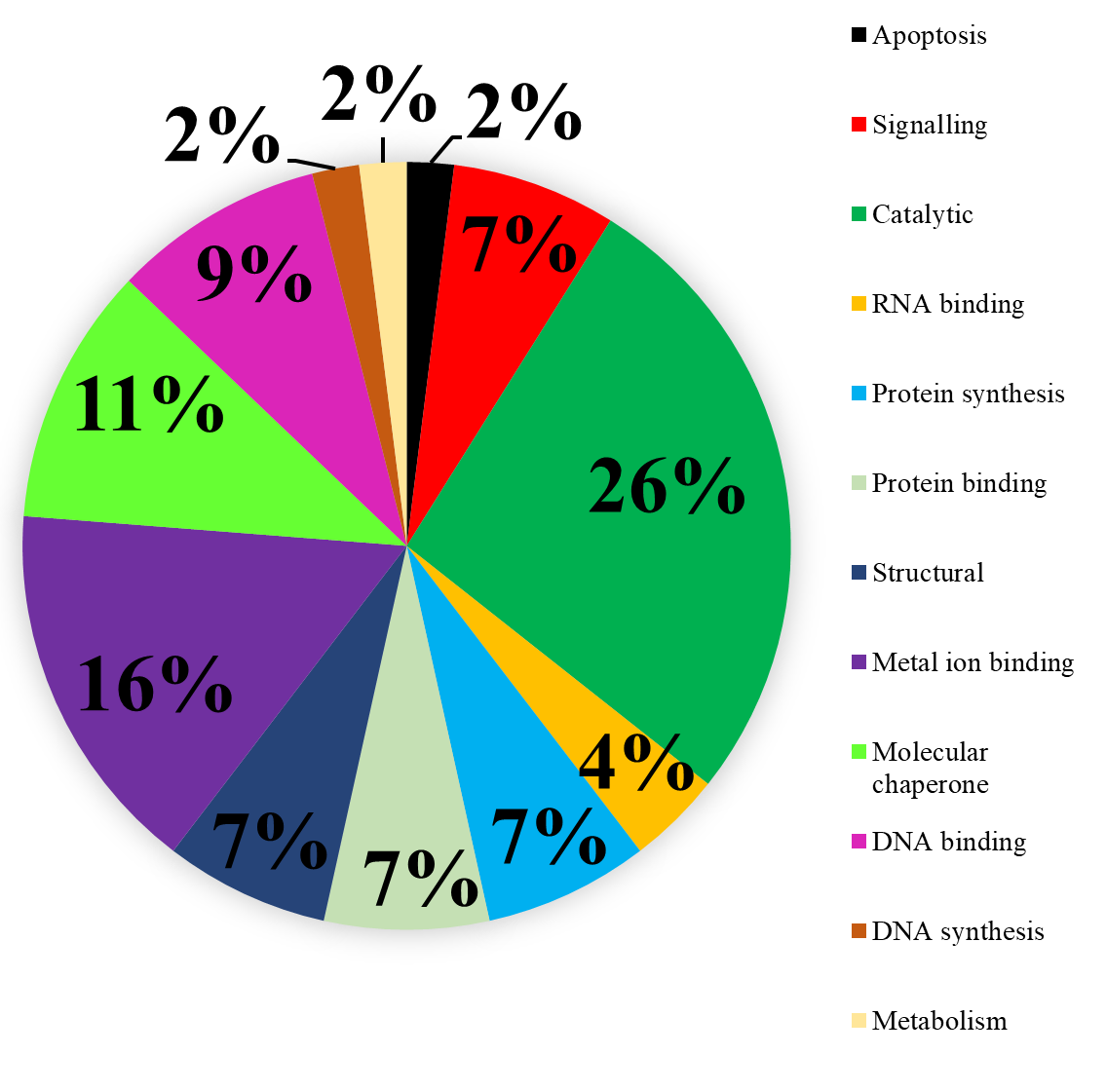 | 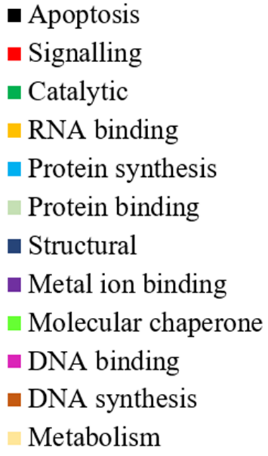 | 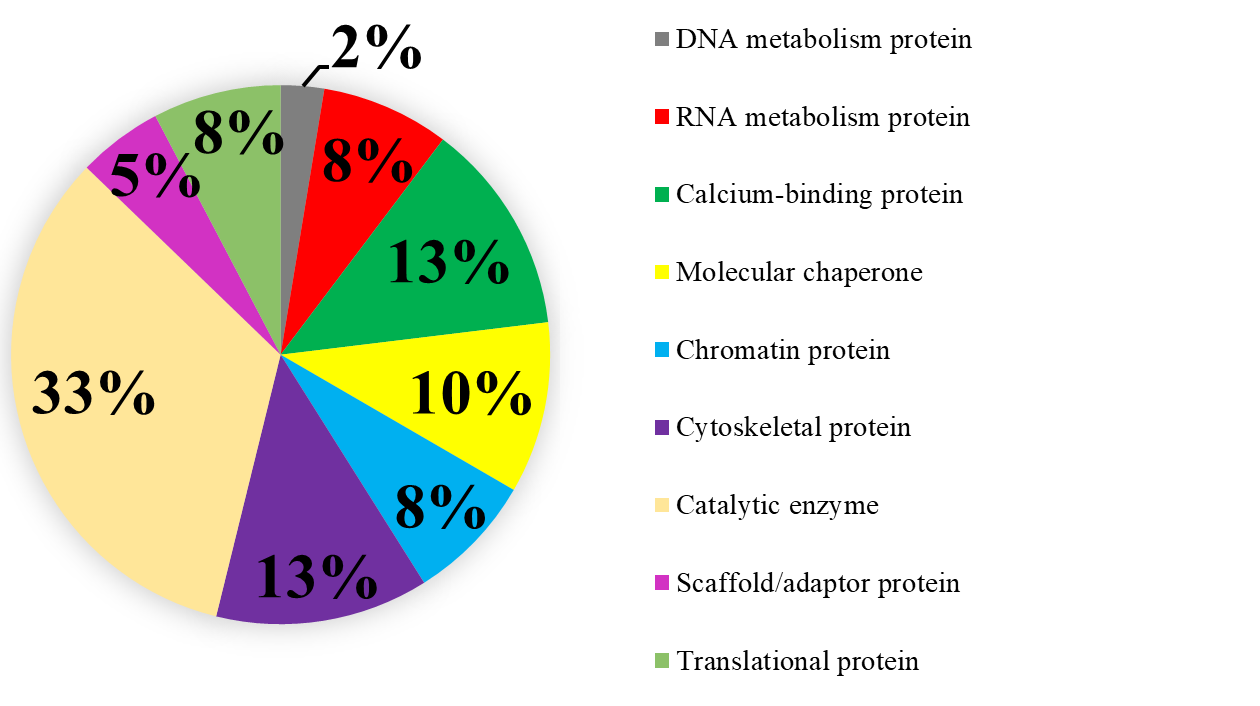 | 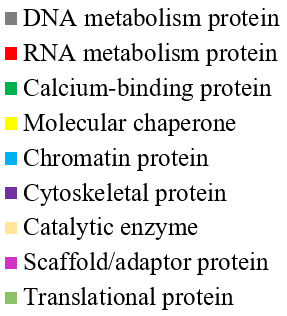 |
| **(c) Subcellular localisation** | | **FIGURE 1:** Functional classifications of the identified proteins in MCF-7 breast cancer cells compared to MCF10A breast normal epithelial cells without zinc exposure (T_0_). The pie charts demonstrate the distributions of the identified proteins in MCF-7 cells compared to MCF10A cells at T_0_ based on (a) Molecular functions (obtained from literature survey and UniProt database) (b) Protein classes (categorized using PANTHER database) and (c) Sub-cellular localisations (derived from literature review and UniProt database). | |
| **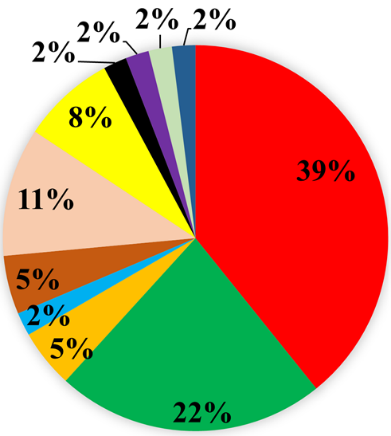** | **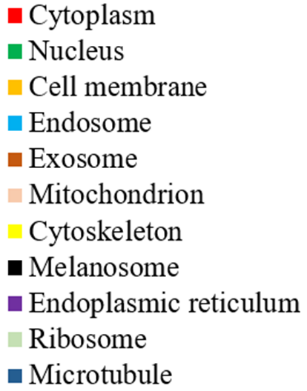** |  |  |

| **(a) Molecular function** | | **(b) Protein class** | |
| --- | --- | --- | --- |
| 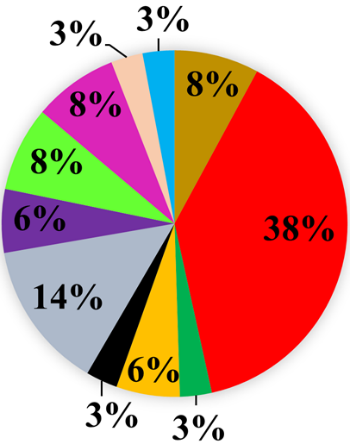 | 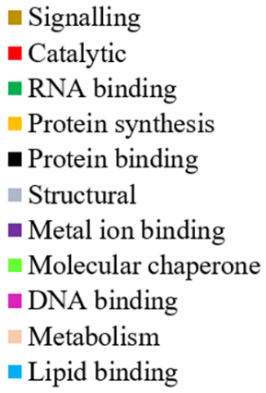 | 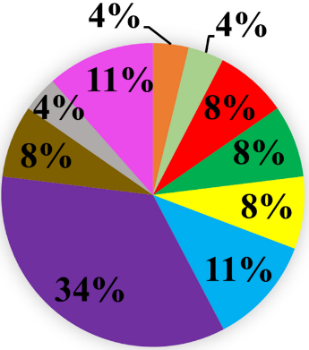 | 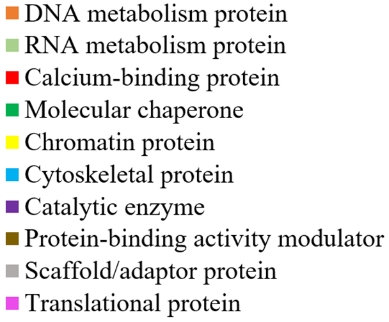 |
| **(c) Subcellular localisation** | | **FIGURE 2:** Functional classifications of the identified proteins in MCF-7 breast cancer cells compared to MCF10A breast normal epithelial cells under zinc exposure for T_120_. The pie charts demonstrate the distributions of the identified proteins in MCF-7 cells compared to MCF10A cells with zinc exposure based on (a) Molecular functions (obtained from literature survey and UniPro database) (b) Protein classes (categorized using PANTHER database) and (c) Subcellular localisations (derived from literature review and UniProt database). | |
| 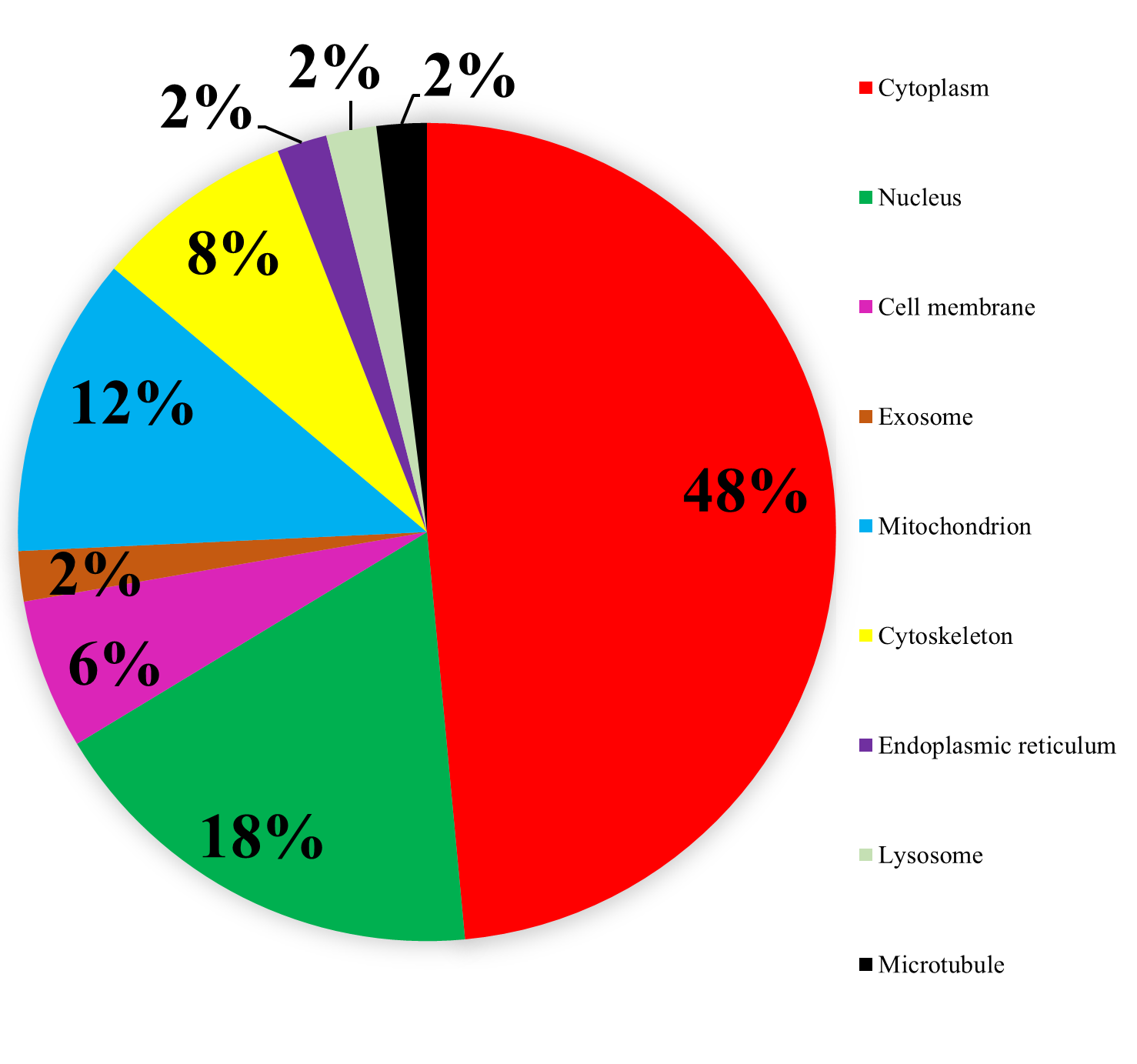 | 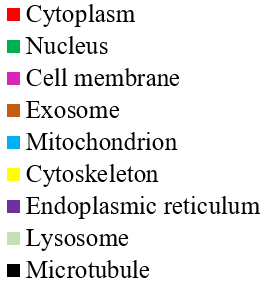 |  |  |

| **(a) Molecular function** | | **(b) Protein class** | |
| --- | --- | --- | --- |
| 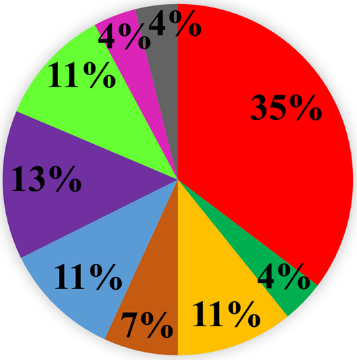 | 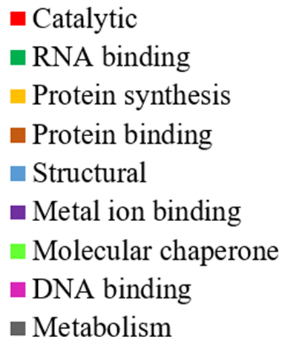 | 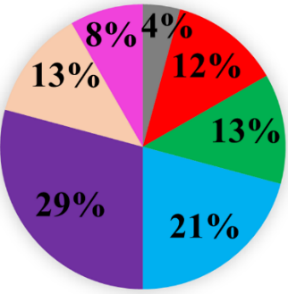 | 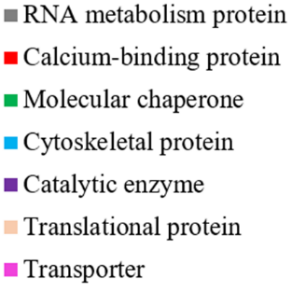 |
| **(c) Subcellular localisation** | | **FIGURE 3:** Functional classifications of the identified proteins in MCF-7 breast cancer cells with exogenous zinc exposure for 120 min (T_120_) compared to without zinc exposure (T_0_). The pie charts demonstrate the distributions of the identified proteins in MCF-7 cells based on (a) Molecular functions (based on literature survey and UniProt database) (b) Protein classes (categorized using PANTHER database) and (c) Subcellular localisations (based on literature review and UniProt database). | |
| **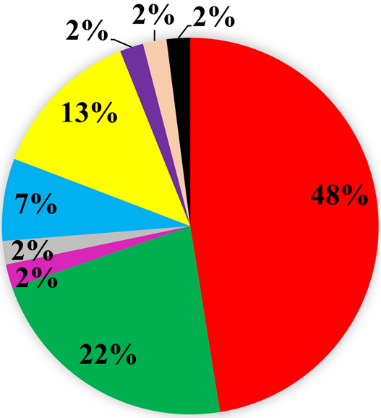** | **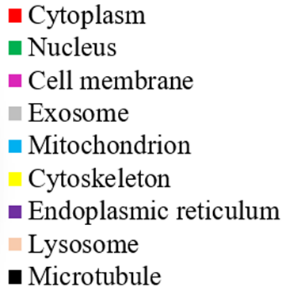** |  |  |

| **(a) Molecular function** | | **(b) Protein class** | |
| --- | --- | --- | --- |
| 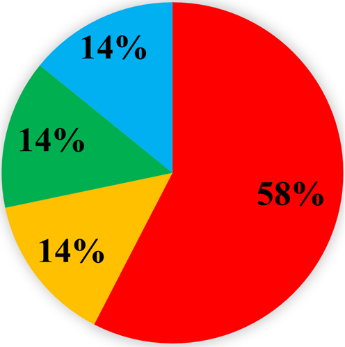 | 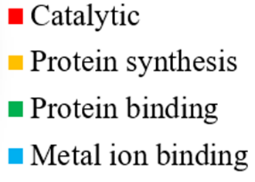 | 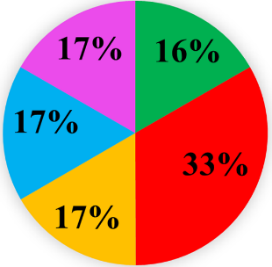 | 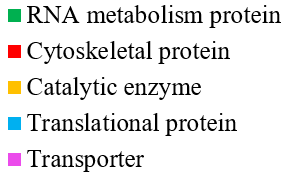 |
| **(c) Subcellular localisation** | | **FIGURE 4:** Functional classifications of the identified proteins in MCF10A breast normal epithelial cells with exogenous zinc exposure for 120 min (T_120_) compared to without zinc exposure (T_0_). The pie charts demonstrate the distributions of the identified proteins in MCF10A cells based on (a) Molecular functions (obtained from literature survey and UniProt database) (b) Protein classes (categorized using PANTHER database) and (c) Subcellular localisations (derived from literature review and UniProt database). | |
| **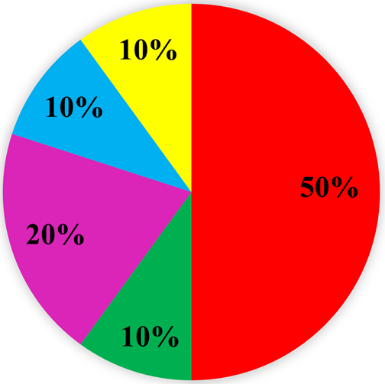** | **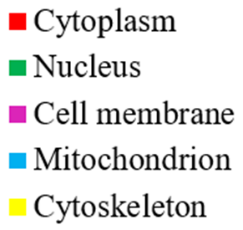** |  |  |

| **(a) Molecular function** | | **(b) Protein class** | |
| --- | --- | --- | --- |
| 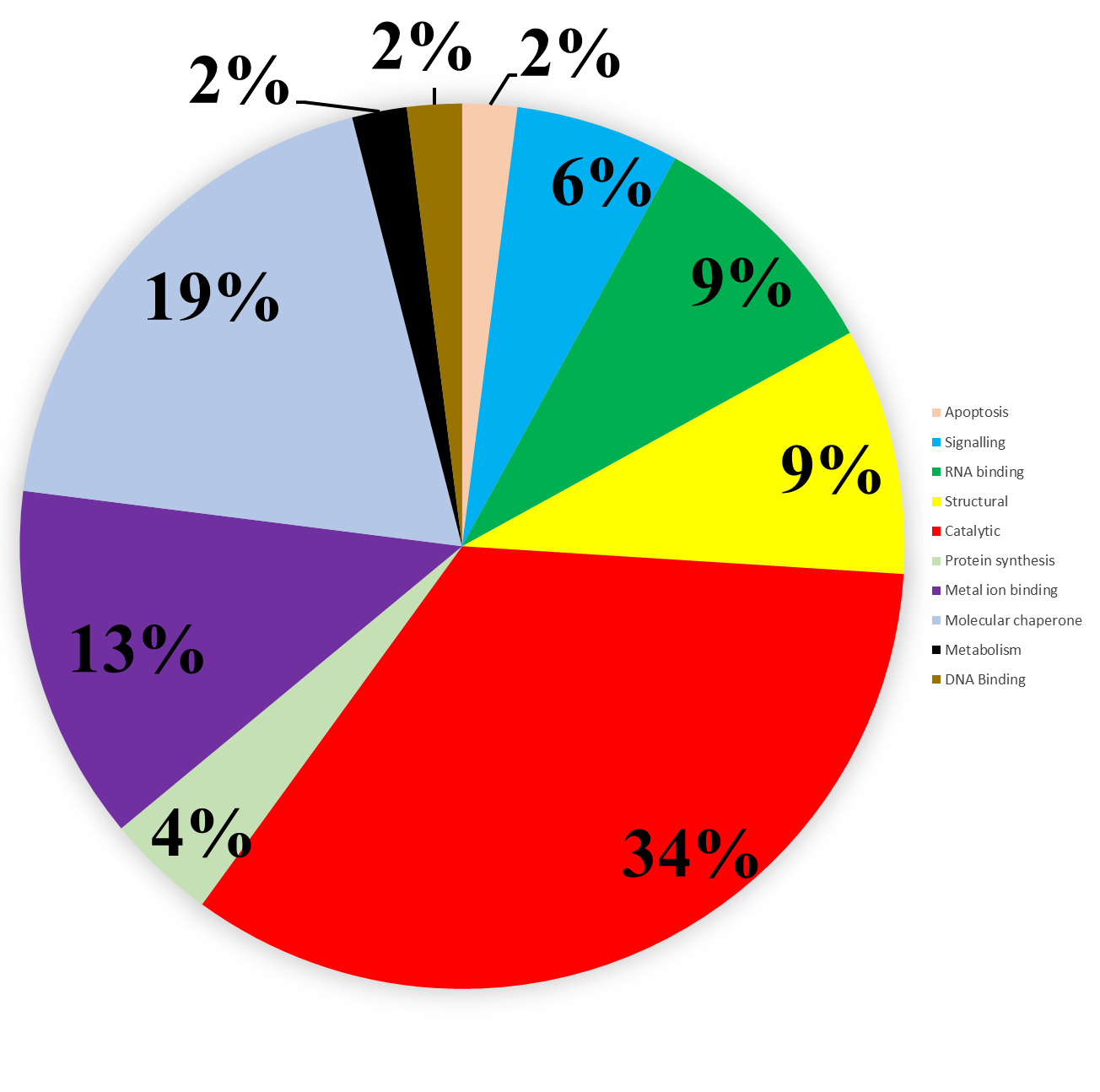 | 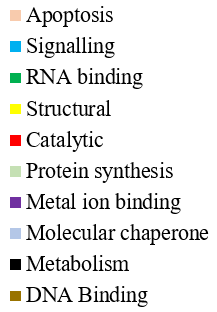 | 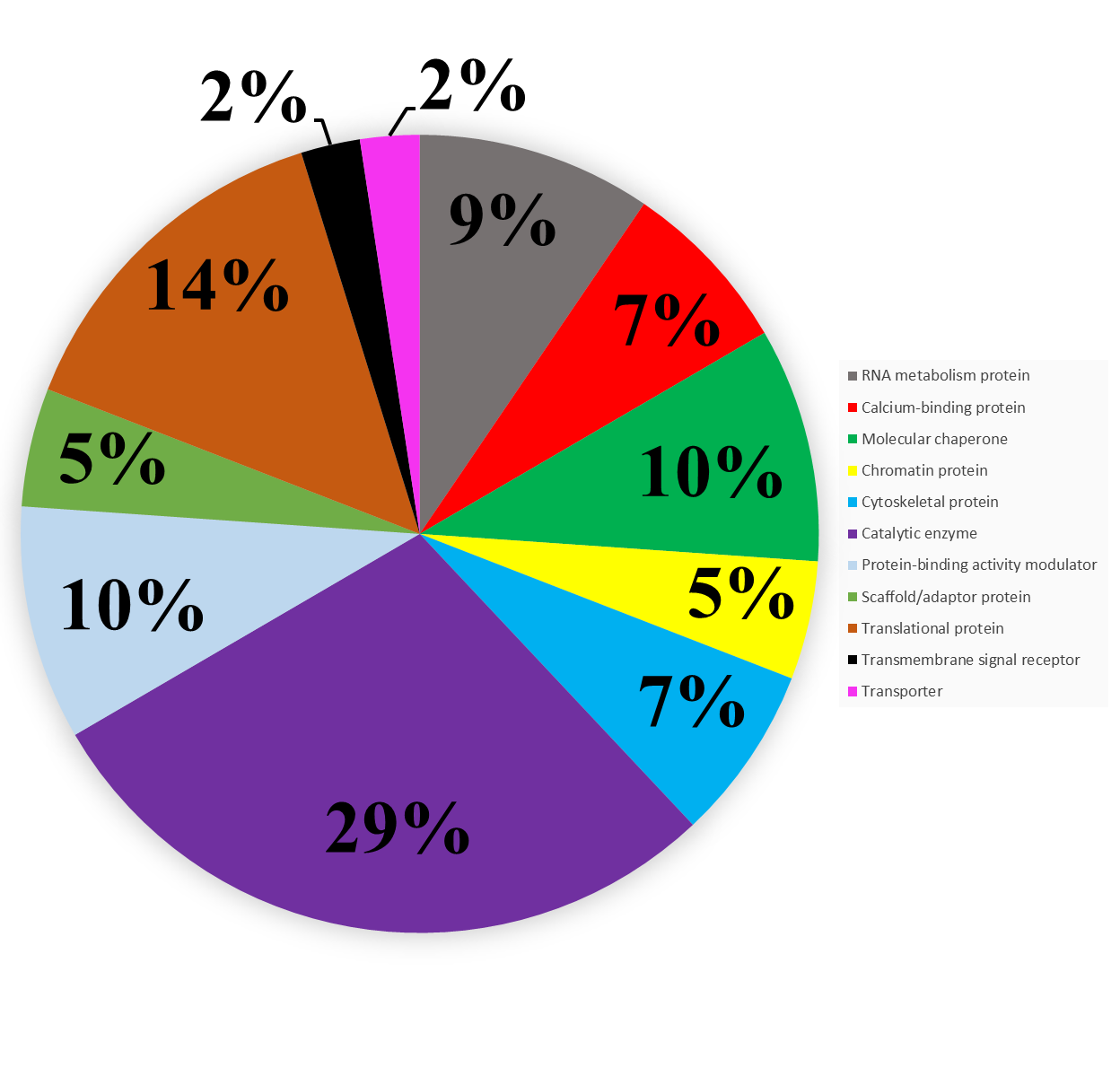 | 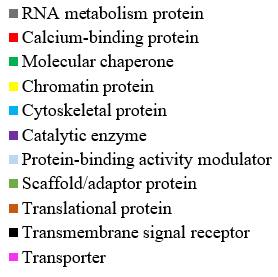 |
| **(c) Subcellular localisation** | | **FIGURE 5:** Functional classifications of the identified proteins in PC3 prostate cancer cells compared to RWPE-1 prostate normal epithelial cells without zinc exposure (T_0_). The pie charts demonstrate the distributions of the identified proteins in PC3 cells compared to RWPE-1 cells without zinc exposure based on (a) Molecular functions (obtained from literature survey and UniProt database) (b) Protein classes (categorized using PANTHER database) and (c) Subcellular localisations (derived from literature review and UniProt database). | |
| 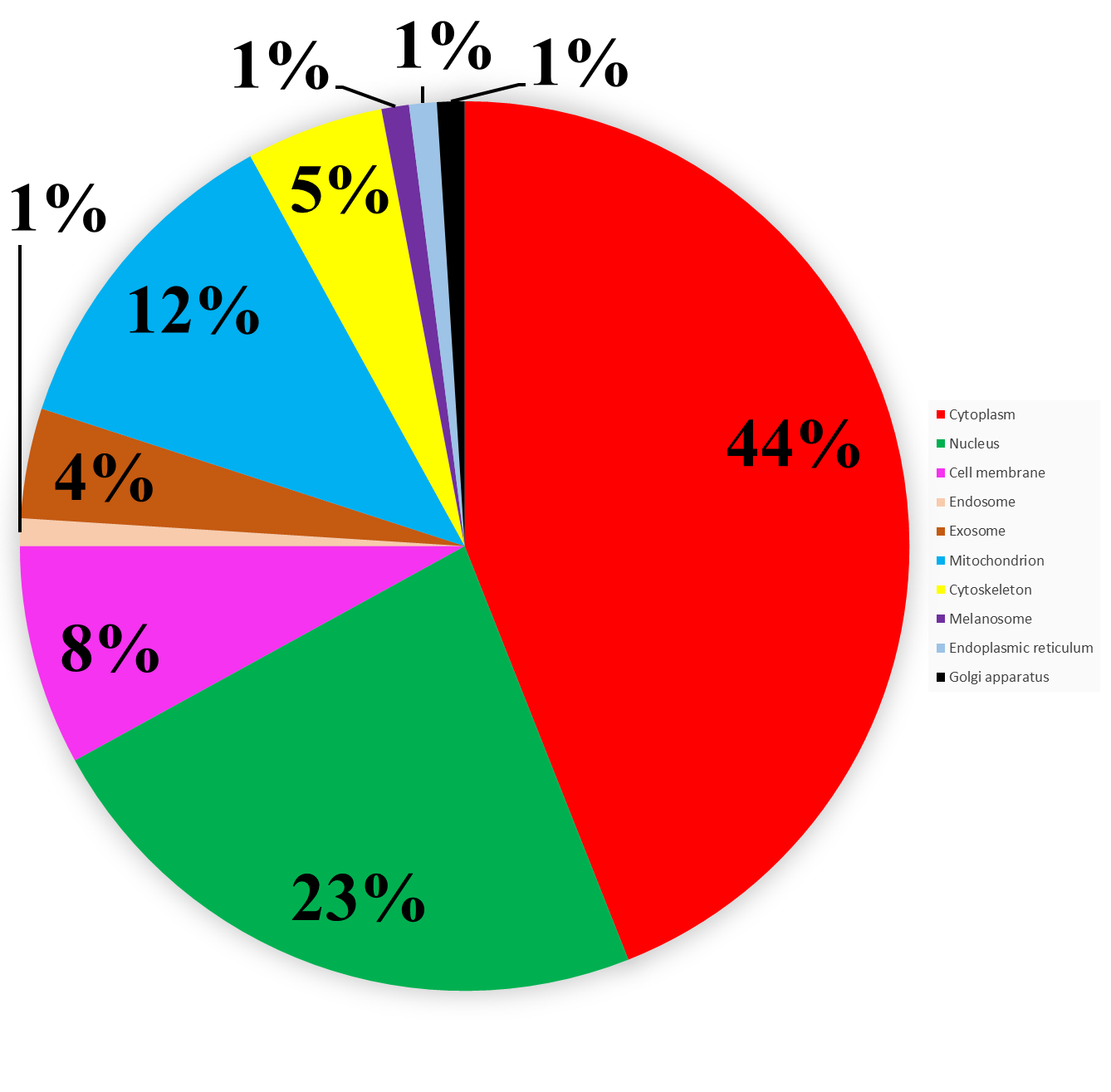 | 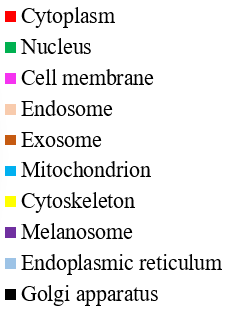 |  |  |

| **(a) Molecular function** | | **(b) Protein class** | |
| --- | --- | --- | --- |
| 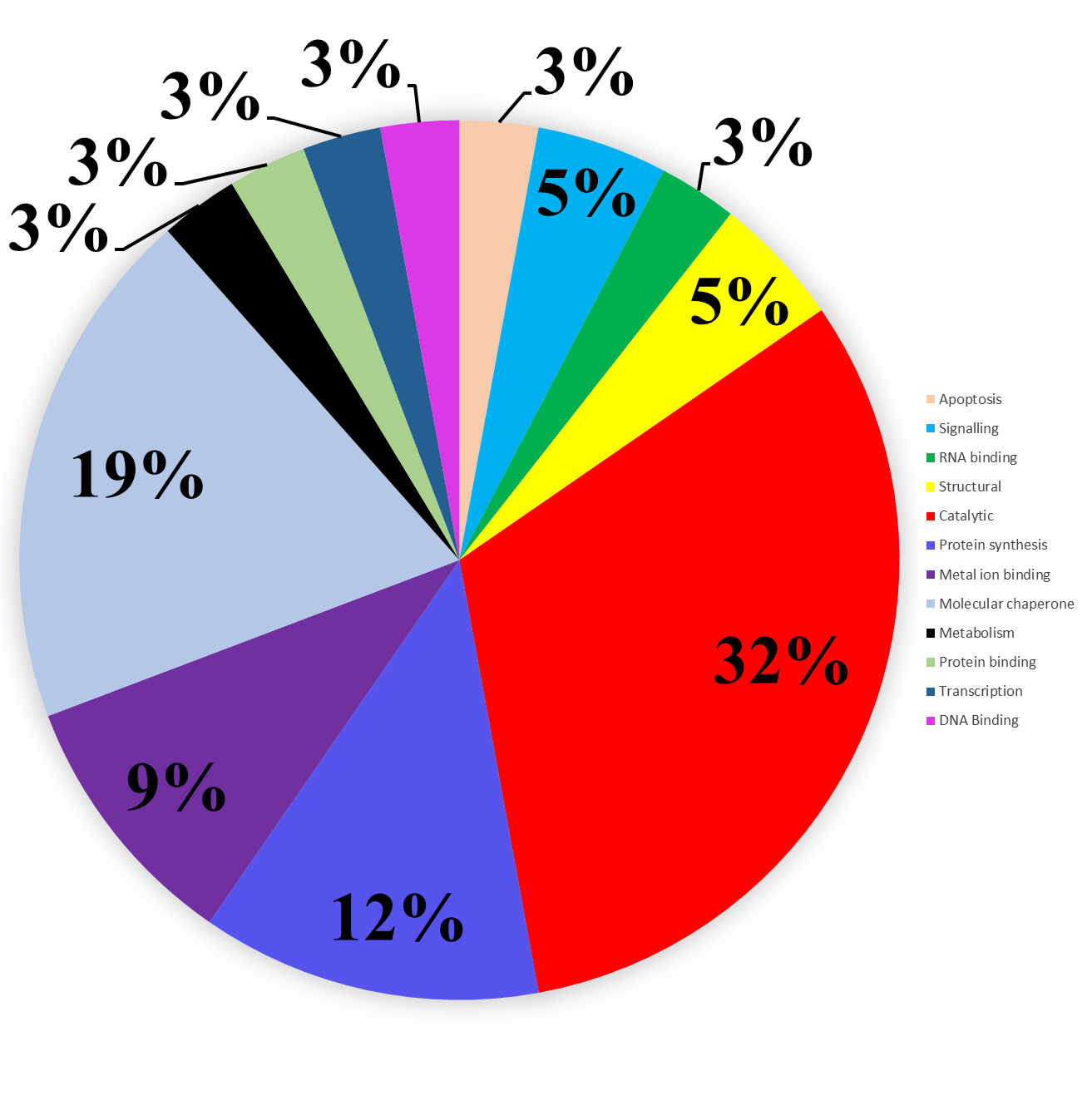 | 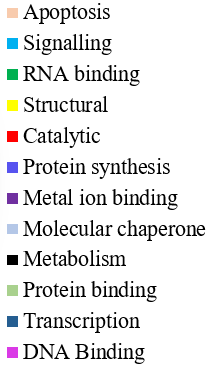 | 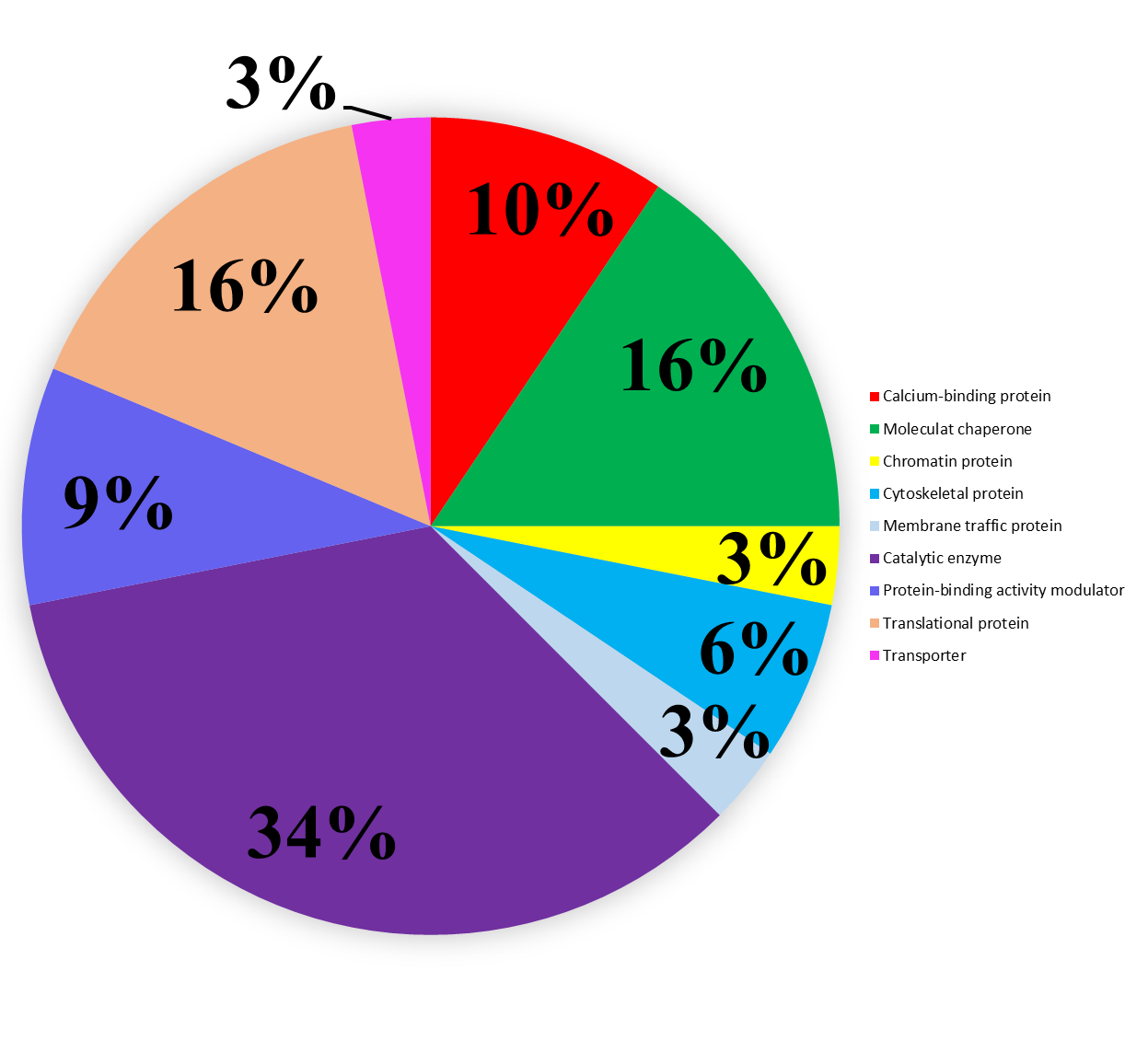 | 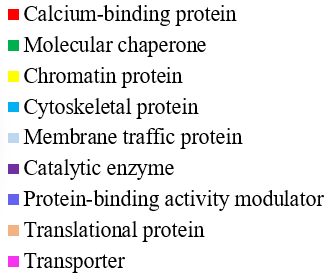 |
| **(c) Subcellular localisation** | | **FIGURE 6:** Functional classifications of the identified proteins in PC3 prostate cancer cells compared to RWPE-1 prostate normal epithelial cells under zinc exposure for 120 min (T_120_). The pie charts demonstrate the distributions of the identified proteins in PC3 cells compared to RWPE-1 cells with zinc exposure based on (a) Molecular functions (obtained from literature survey and UniProt database) (b) Protein classes (categorized using PANTHER database) and (c) Subcellular localisations (derived from literature review and UniProt database). | |
| 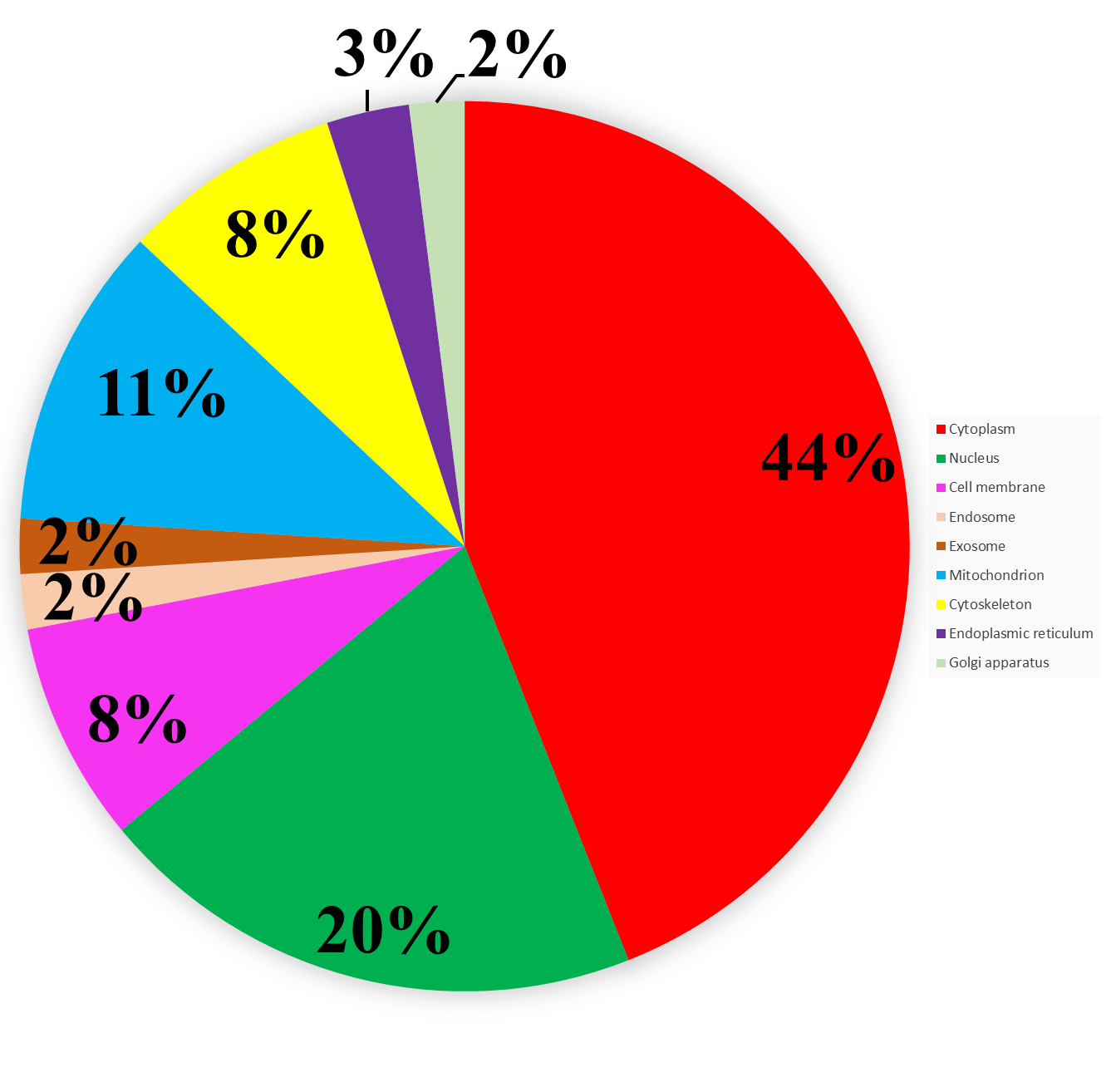 | 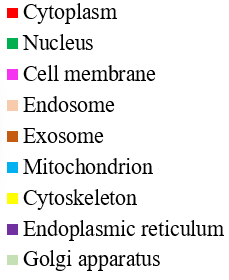 |  |  |

| **(a) Molecular function** | | **(b) Protein class** | |
| --- | --- | --- | --- |
| 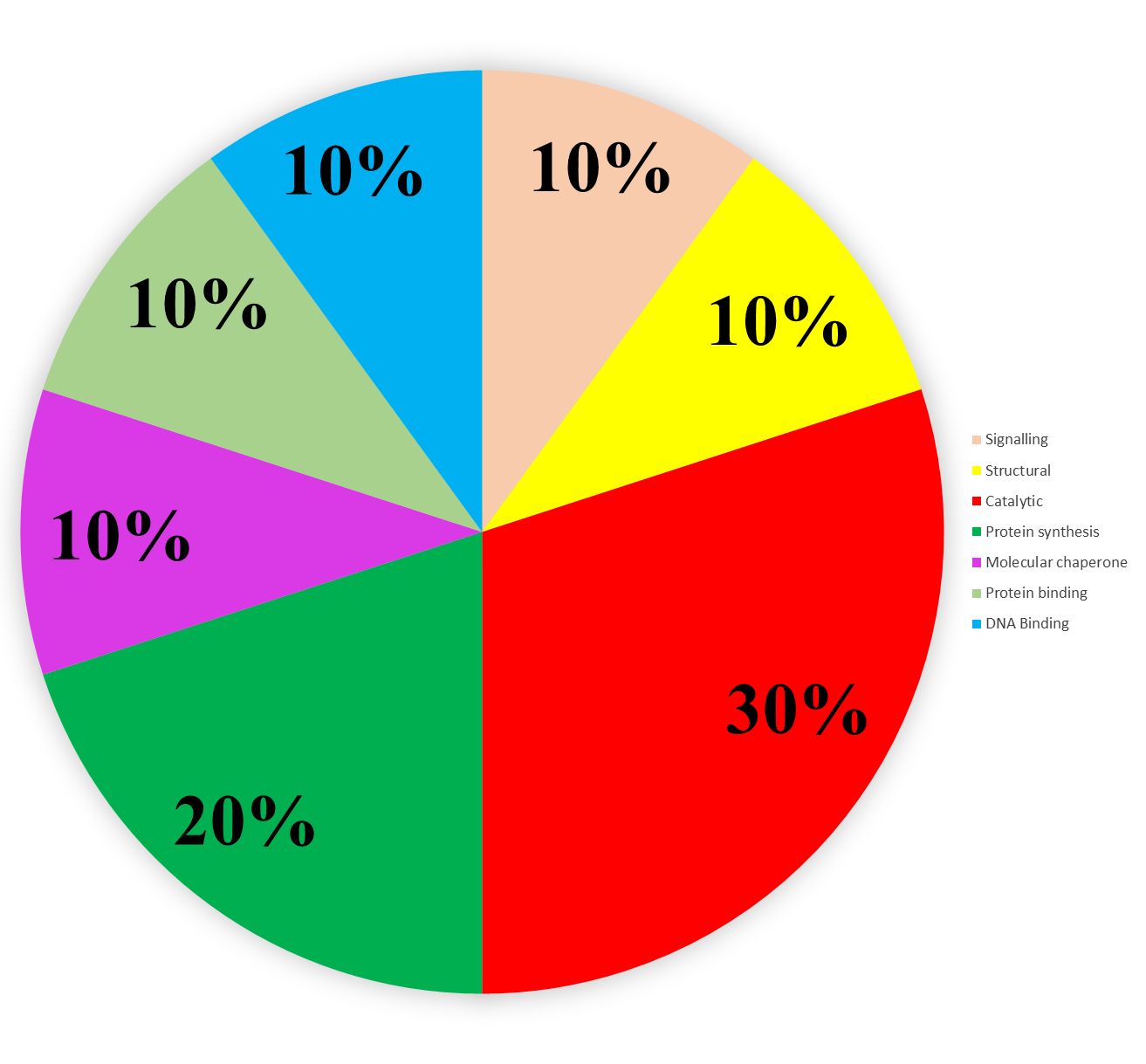 | 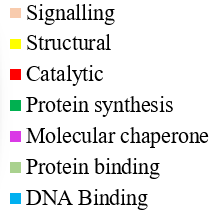 | 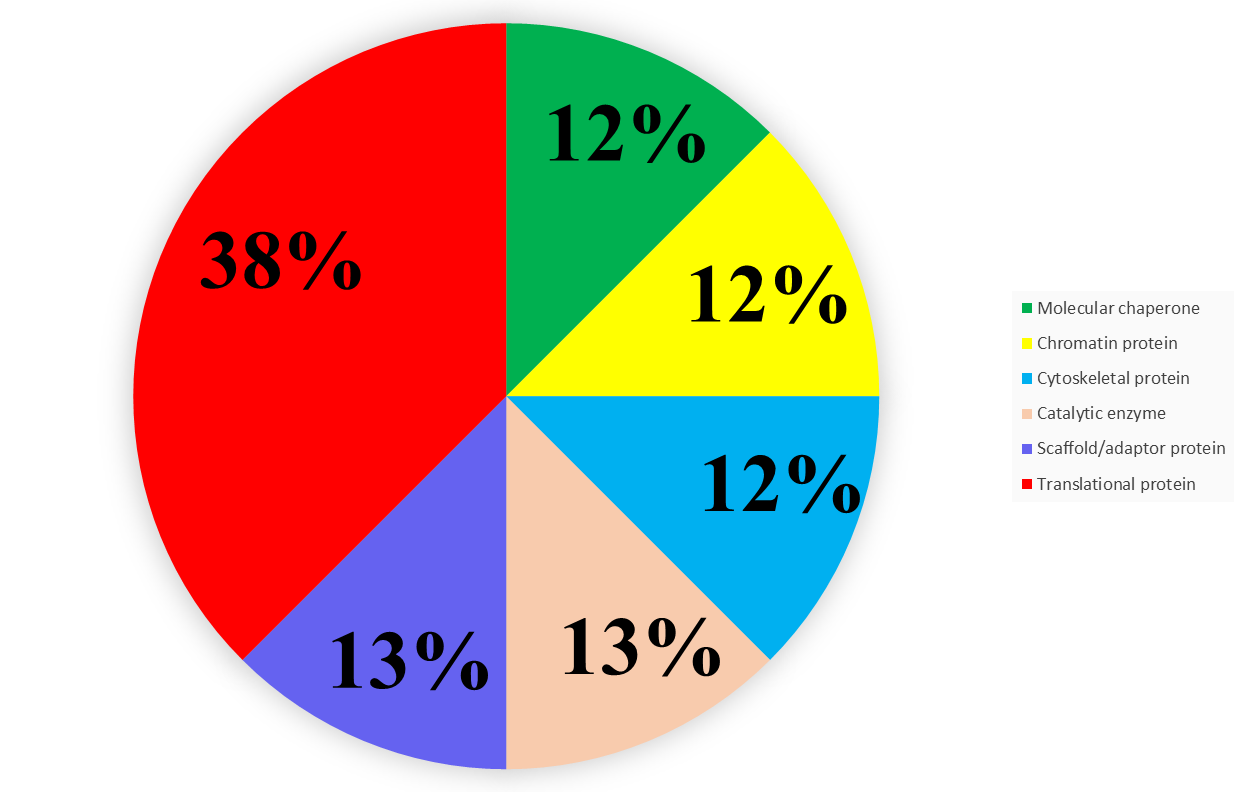 | 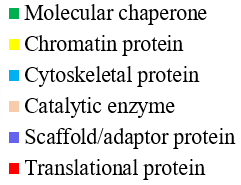 |
| **(c) Subcellular localisation** | | **FIGURE 7:** Functional classifications of the identified proteins in PC3 prostate cancer cells with exogenous zinc exposure for 120 min (T_120_) compared to without zinc exposure (T_0_). The pie charts demonstrate the distributions of the identified proteins in PC3 cells based on (a) Molecular functions (obtained from literature survey and UniProt database) (b) Protein classes (categorized using PANTHER database) and (c) Subcellular localisations (derived from literature review and UniProt database). | |
| 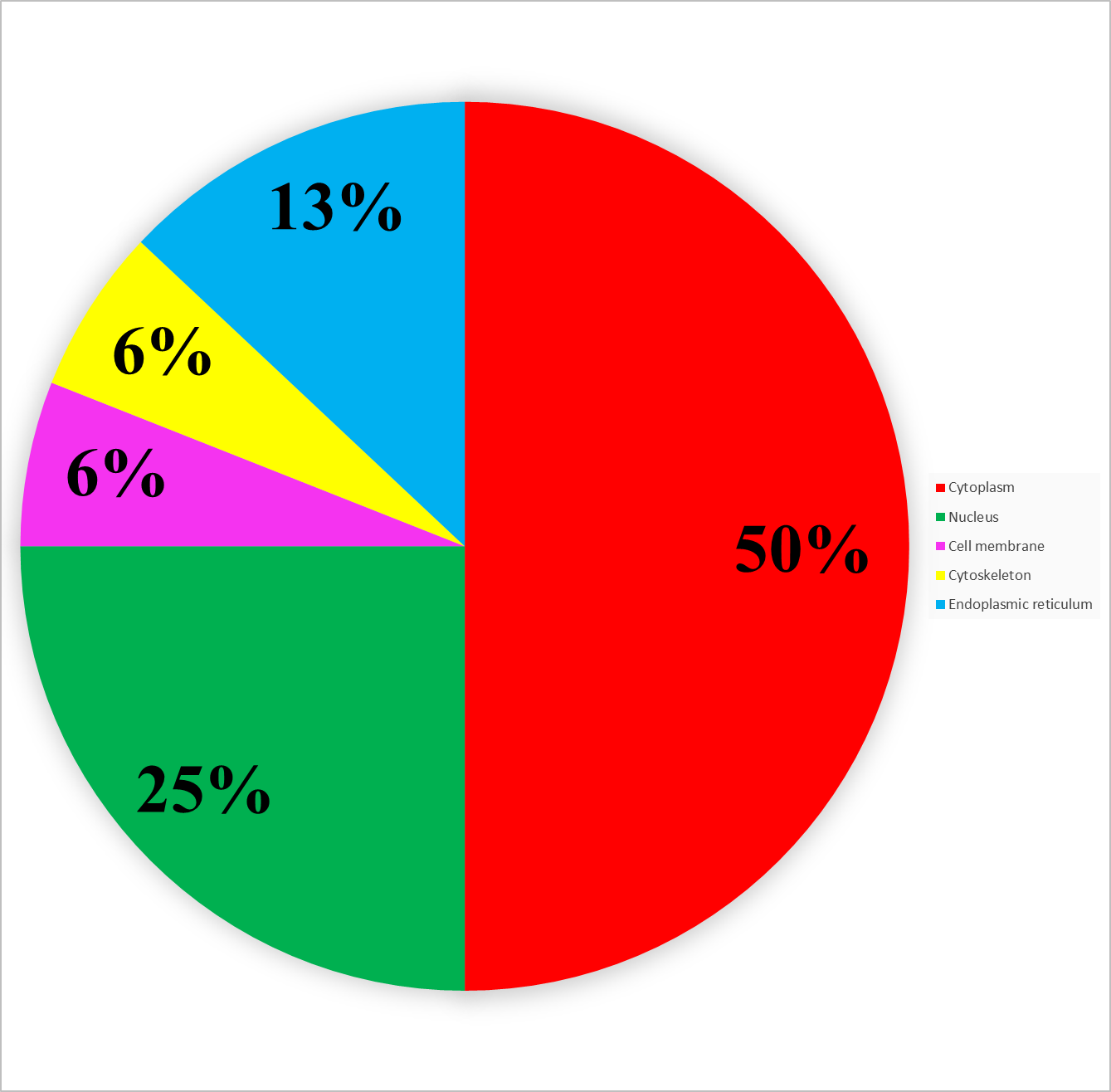 | 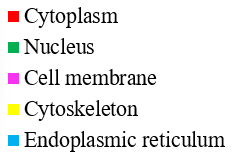 |  |  |

| **(a) Molecular function** | | **(b) Protein class** | |
| --- | --- | --- | --- |
| 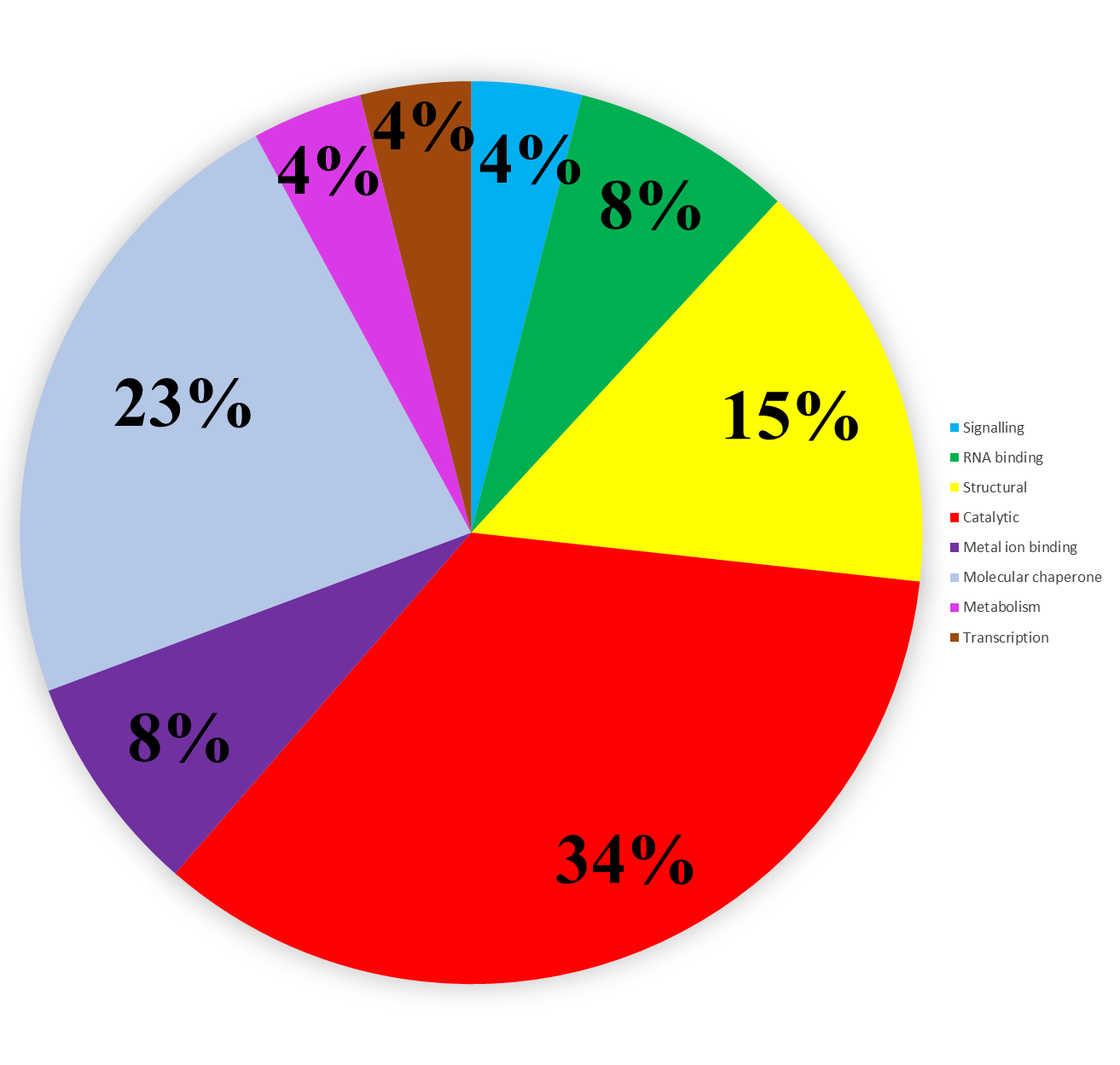 | 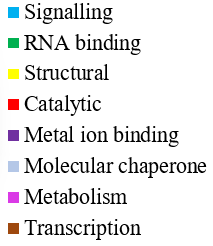 | 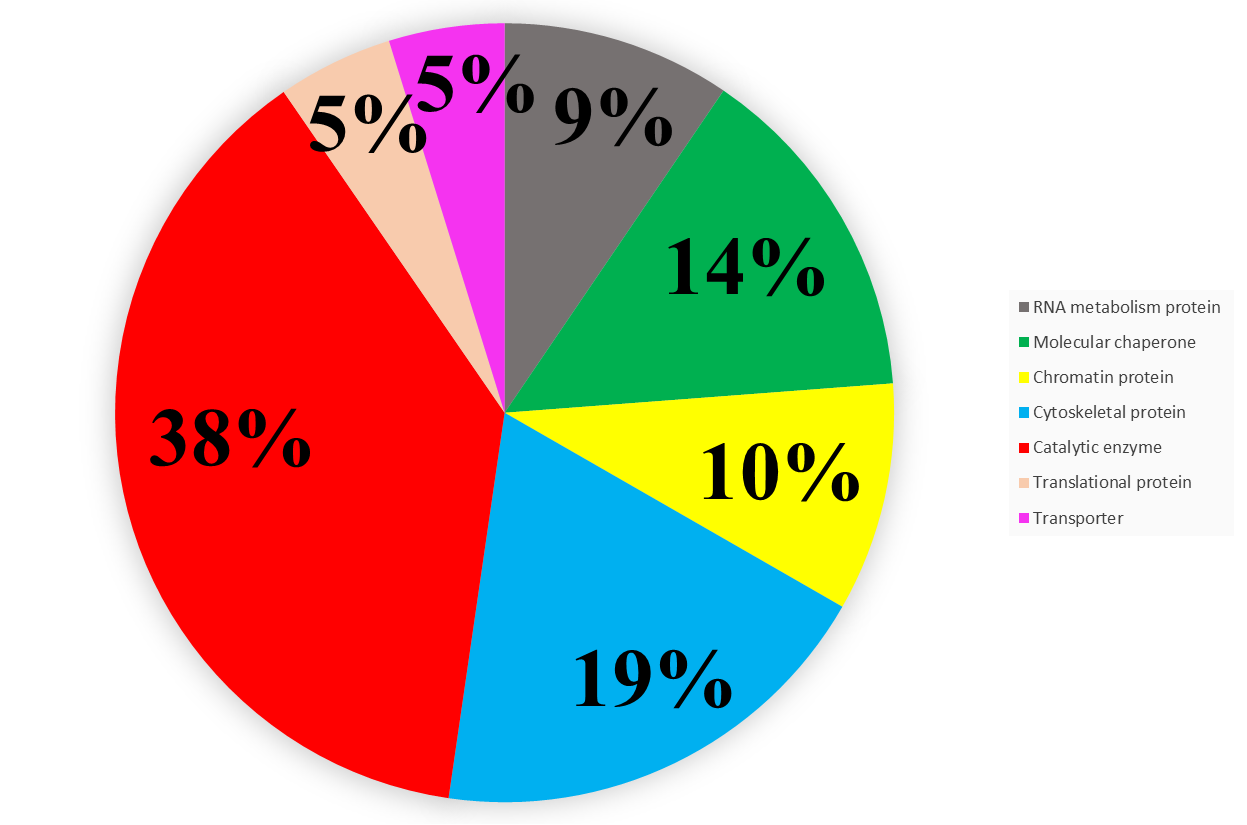 | 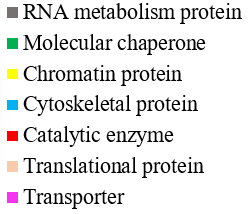 |
| **(c) Subcellular localisation** | | **FIGURE 8:** Functional classifications of the identified proteins in RWPE-1 prostate normal epithelial cells with exogenous zinc exposure for 120 min (T_120_) compared to without zinc exposure (T_0_). The pie charts demonstrate the distributions of the identified proteins in RWPE-1 cells based on (a) Molecular functions (obtained from literature survey and UniProt database) (b) Protein classes (categorized using PANTHER database) and (c) Subcellular localisations (derived from literature review and UniProt database). | |
| 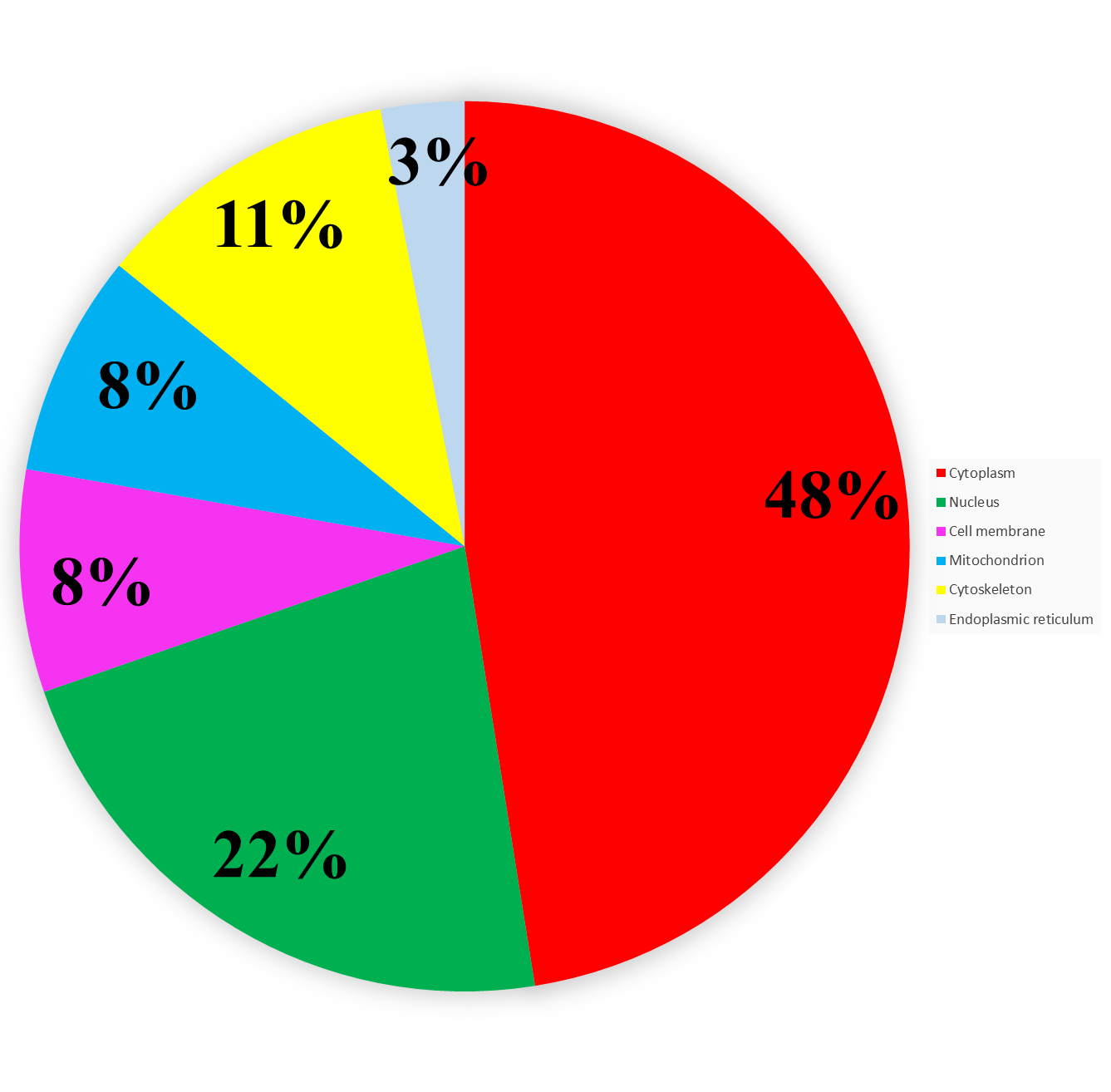 | 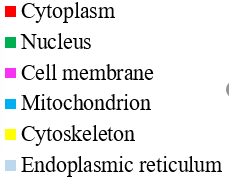 |  |  |

| **(a) MCF-7 T_0_ vs MCF10A T_0_** | | **(c) MCF-7 T_120_ vs MCF-7 T_0_** | |
| --- | --- | --- | --- |
| 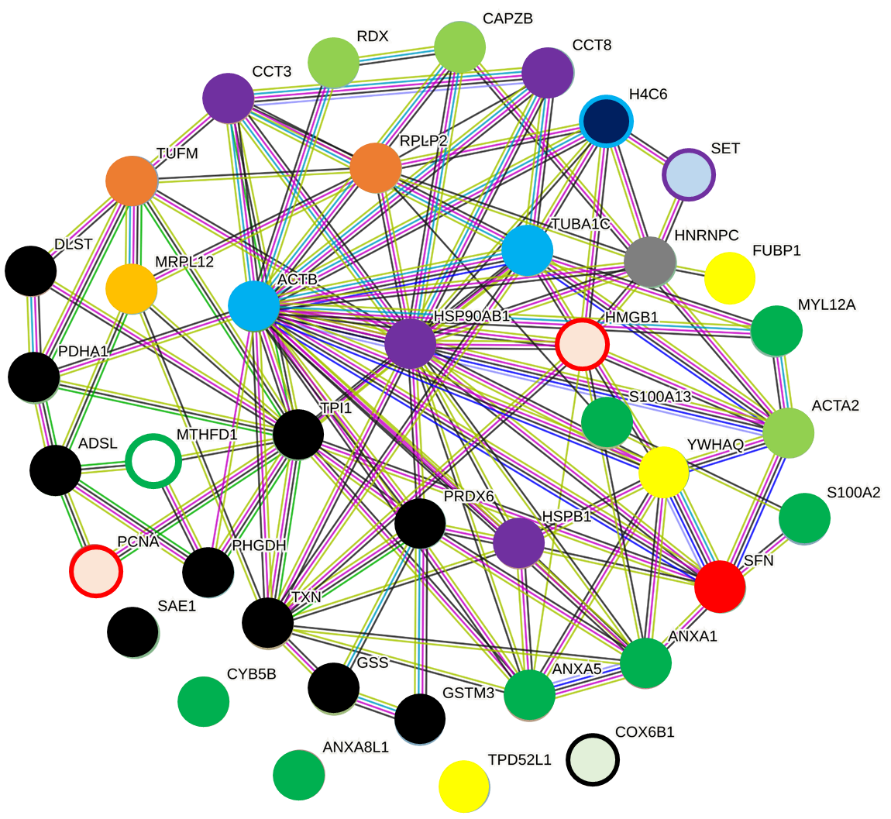 | | 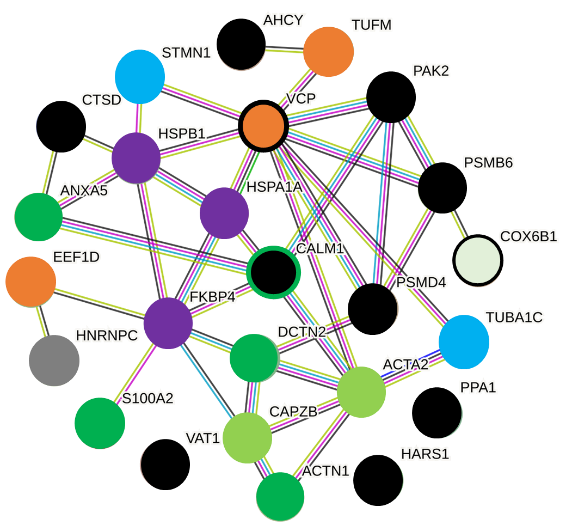 | |
|  |  | **(d) MCF10A T_120_ vs MCF10A T_0_** | |
|  |  | 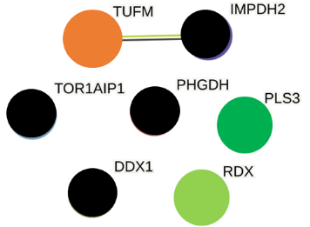 | |
| **(b) MCF-7 T_120_ vs MCF10A T_120_** | 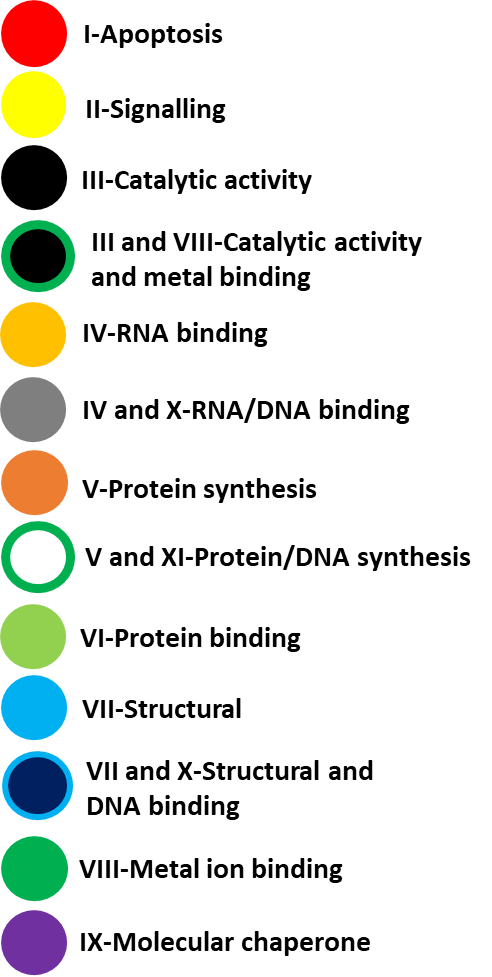 | | **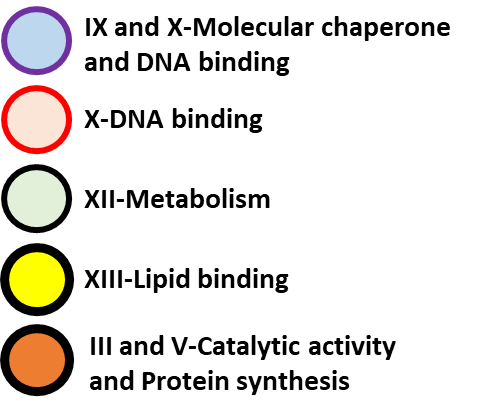** |
| 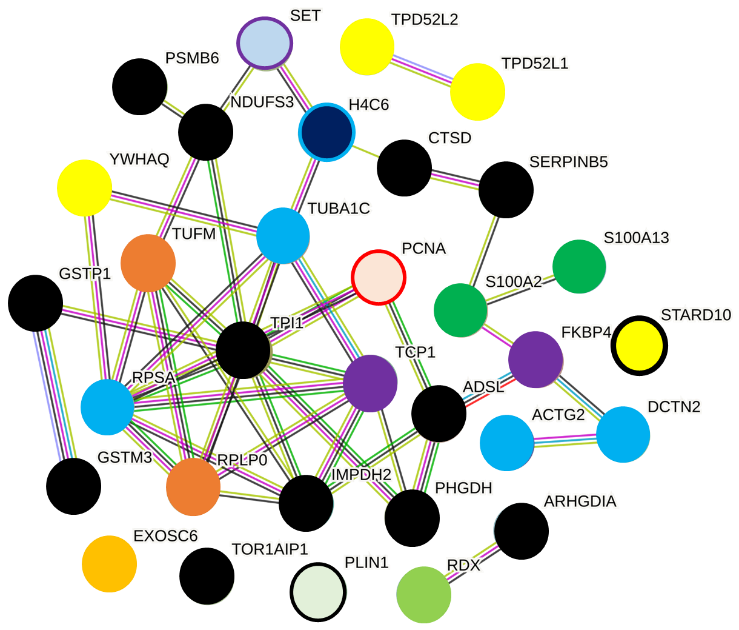 |  |  |  |
| **FIGURE 9:** Functional interactions of the differentially expressed proteins in breast cancerous MCF-7 cells and breast normal epithelial MCF10A cells without and with exogenous zinc exposure by the STRING analysis. (a) Functional interactions of the differentially expressed proteins in MCF-7 cells compared to MCF10A cell without exogenous zinc exposure (T_0_), (b) Functional interactions of the differentially expressed proteins in MCF-7 cells compared to MCF10A cell under exogenous zinc exposure for 120 min (T_120_), (c) Functional interactions of the differentially expressed proteins in MCF-7 cells at T_120_ compared to T_0_, and (d) Functional interactions of the differentially expressed proteins in MCF10A cells at T_120_ compared to T_0_. The differentially expressed proteins perform a wide range of functions including apoptosis, signalling, catalytic activity, RNA binding, protein synthesis, protein binding, structural, metal ion binding, molecular chaperone, DNA binding, DNA synthesis, metabolism, and lipid binding. The different colour lines indicate the type of interaction evidence. The known protein interactions determined from curated databases are shown by cyan lines. The known protein interactions determined from the experiments are shown by purple lines. The predicted interactions by green lines for gene neighbourhood, red lines for gene fusions and dark blue lines for gene co-occurrence, while others are shown by yellow lines for text mining, black lines for co-expression and light blue lines for protein homology. | | | |

| **(a) PC3 T_0_ vs RWPE-1 T_0_** | **(c) PC3 T_120_ vs PC3 T_0_** |
| --- | --- |
| 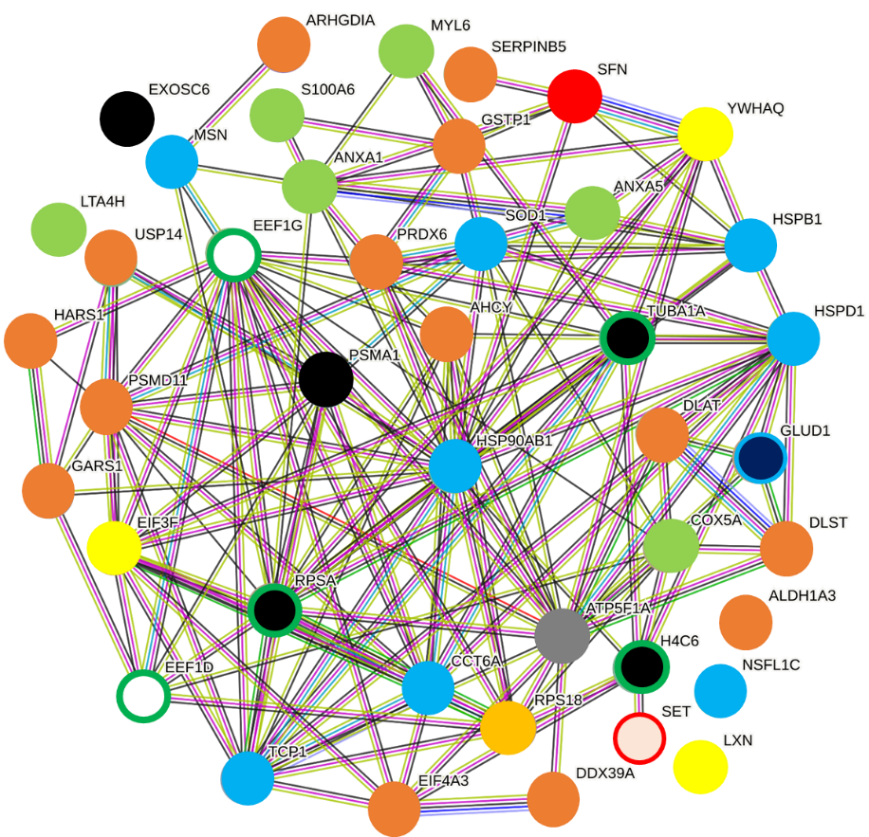 | 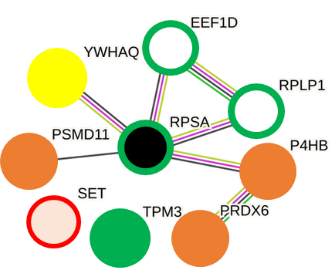 |
|  | **(d) RWPE-1 T_120_ vs RWPE-1 T_0_** |
|  | 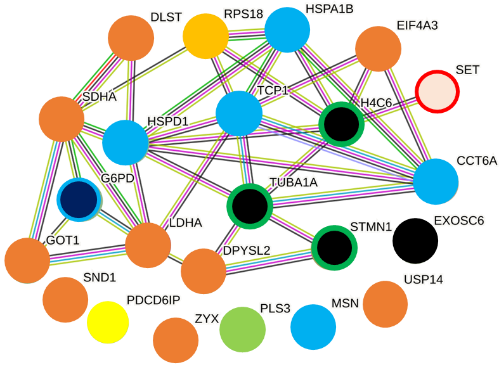 |
|  | **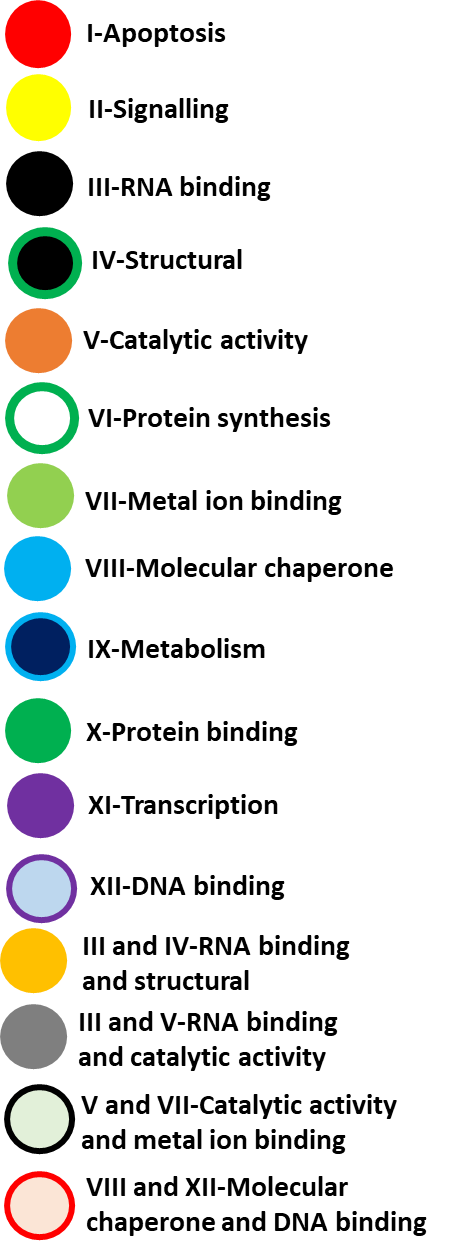** |
| **(b) PC3 T_120_ vs RWPE-1 T_120_** |  |
| 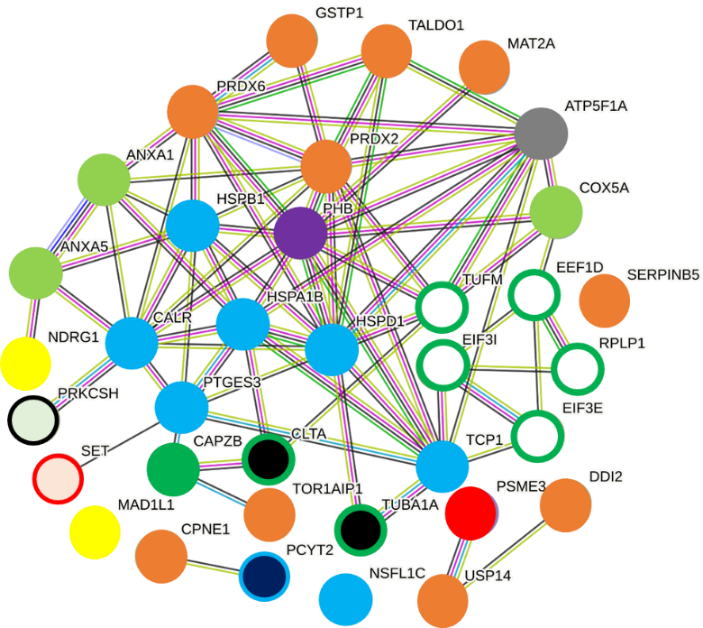 |  |
| **FIGURE 10:** Functional interactions of the differentially expressed proteins in prostate cancerous PC3 cells and prostate normal epithelial RWPE-1 cells without and with exogenous zinc exposure by the STRING analysis. (a) Functional interactions of the differentially expressed proteins in PC3 cells compared to RWPE-1 cells without exogenous zinc exposure (T_0_), (b) Functional interactions of the differentially expressed proteins in PC3 cells compared to RWPE-1 cells under exogenous zinc exposure for 120 min (T_120_), (c) Functional interactions of the differentially expressed proteins in PC3 cells at T_120_ compared to T_0_, and (d) Functional interactions of the differentially expressed proteins in RWPE-1 cells at T_120_ compared to T_0_. The differentially expressed proteins perform a wide range of functions including apoptosis, signalling, RNA binding, structural, catalytic activity, protein synthesis, metal ion binding, molecular chaperone, metabolism, protein binding, transcription, and DNA binding. The different colour lines indicate the type of interaction evidence. The known protein interactions determined from curated databases are shown by cyan lines. The known protein interactions determined from the experiments are shown by purple lines. The predicted interactions by green lines for gene neighbourhood, red lines for gene fusions and dark blue lines for gene co-occurrence, while others are shown by yellow lines for text mining, black lines for co-expression and light blue lines for protein homology. | |
